# Assessment of indoor air quality in health clubs: insights into (ultra)fine and coarse particles and gaseous pollutants

**DOI:** 10.3389/fpubh.2023.1310215

**Published:** 2023-11-28

**Authors:** Cátia Peixoto, Maria do Carmo Pereira, Simone Morais, Klara Slezakova

**Affiliations:** ^1^REQUIMTE–LAQV, Instituto Superior de Engenharia do Porto, Instituto Politécnico do Porto, Rua Dr. António Bernardino de Almeida, Porto, Portugal; ^2^LEPABE-ALiCE, Faculdade de Engenharia da Universidade do Porto, Rua Dr. Roberto Frias, Porto, Portugal

**Keywords:** indoor air, gaseous pollutants, particulate matter, particle number concentration, comfort parameters, health clubs, physical exercise

## Abstract

**Introduction:**

Exercising on regular basis provides countless health benefits. To ensure the health, well-being and performance of athletes, optimal indoor air quality, regular maintenance and ventilation in sport facilities are essential.

**Methods:**

This study assessed the levels of particulate, down to the ultrafine range (PM_10_, PM_2.5_, and particle number concentration in size range of 20–1,000 nm, i.e., – PNC_20-1000 nm_), gaseous pollutants (total volatile organic compounds – TVOCs, CO_2_, and O_3_) and comfort parameters (temperature – T, relative humidity – RH) in different functional spaces of health clubs (*n* = 8), under specific occupancy and ventilation restrictions.

**Results and Discussion:**

In all HCs human occupancy resulted in elevated particles (up to 2–3 times than those previously reported), considering mass concentrations (PM_10_: 1.9–988.5 μg/m^3^ PM_2.5_: 1.6–479.3 μg/m^3^) and number (PNC 1.23 × 10^3^ – 9.14 × 10^4^ #/cm^3^). Coarse and fine PM indicated a common origin (r_s_ = 0.888–0.909), while PNC showed low–moderate associations with particle mass (r_s_ = 0.264–0.629). In addition, up to twice-higher PM and PNC were detected in cardiofitness & bodybuilding (C&B) areas as these spaces were the most frequented, reinforcing the impacts of occupational activities. In all HCs, TVOCs (0.01–39.67 mg/m^3^) highly exceeded the existent protection thresholds (1.6–8.9 times) due to the frequent use of cleaning products and disinfectants (2–28 times higher than in previous works). On contrary to PM and PNC, TVOCs were higher (1.1–4.2 times) in studios than in C&B areas, due to the limited ventilations combined with the smaller room areas/volumes. The occupancy restrictions also led to reduced CO_2_ (122–6,914 mg/m^3^) than previously observed, with the lowest values in HCs with natural airing. Finally, the specific recommendations for RH and T in sport facilities were largely unmet thus emphasizing the need of proper ventilation procedures in these spaces.

## 1 Introduction

One of the nowadays concerns is related with quality of air (1–4) in indoor spaces, as those are the environment in which people spend majority of their time (5–7). Sport facilities, such as gyms, fitness centers and health clubs represent in this context a unique indoor microenvironment where occupants may face increased exposure to indoor pollutants due to heightened physical activity (8–14). The levels of pollution in these environments depends on human presence (such as breathing, sweating) (3, 15, 16) and the respective physical activities performed (3, 17). In addition, emissions from the particular products and equipment (e.g., plastic and rubber materials in flooring, anti-slip agents, support mats and cushions) or personal care, sanitation and cleaning products (disinfectants and personal care products) (18, 19) can cause additional risk. In that regard, the previous studies emphasized some of the health-relevant gaseous [such as TVOCs, CH_2_O, NO_2_, SO_2_, O_3_, CO, and CO_2_; (12, 13, 20–22)] and particulate pollutants (20, 23–27). However, what concerns the sport facilities, less information is available for different particle fractions or particle metrics (mass vs. number concentration). In sport facilities, adequate ventilation is crucial (28), not only to reduce the risk of exposure to indoor pollutants but also to ensure the removal of sweat and odors (23, 29). In addition, proper ventilation in these spaces is required to regulate the comfort parameters (indoor temperature and humidity) in order to prevent overheating and discomfort during intense physical activities (6, 7, 30). In the post-pandemic era, effective ventilation has gained even greater significance to safeguard the health and well-being of occupants in indoor spaces in general.

The aim of this study was to assess the levels of particulate (PM_10_, PM_2.5_, and particle number concentration – PNC), gaseous pollutants (total volatile organic compounds – TVOCs, CO_2_, and O_3_) and comfort parameters (temperature – T, relative humidity – RH) in health clubs, under specific occupancy and ventilation restrictions.

## 2 Materials and methods

### 2.1 Sites description

The study was carried out at eight health clubs (HC1 – HC8) that belonged to a chain of low-cost fitness centers. All clubs were located in the urban-background and urban-traffic areas of Oporto Metropolitan Area (north of Portugal) where vehicle traffic and local industry are the main emission sources in the respective ambient air (31, 32). Three clubs, specifically HC1, HC3, and HC4 were situated within shopping centers.

In general, all HCs had a similar organization, comprising main area cardiofitness and bodybuilding (C&B) for cardiovascular fitness (with treadmills, rowing and elliptical machine) and bodybuilding training (array of machines and additional equipment). Additionally, they featured studios for group classes (1–3, studios), and dressing rooms with bathroom with associated functions. Typically, all HCs also included a bar area furnished with vending machines, dedicated spaces for physical and nutritional assessment offices, and administrative and support spaces (reception, storage rooms or support for staff). In addition to these standard features, HC2 and HC5 encompassed functional areas for spa and beauty/healthcare services; HC7 included an indoor studio with a hairdresser. Three out of the eight clubs (HC6, HC7, and HC8) included indoor swimming pools, along with the necessary supplementary facilities. More detailed descriptions of all HCs are summarized in Supplementary Table S1 and Figure S1.

The indoor air quality (IAQ) monitoring was conducted in October 2020 – November 2021, which coincided with the post-lockdown period in Portugal. During this period, stringent sanitary recommendations were enforced for sport facilities to mitigate the transmission of infections. These recommendations encompassed various aspects including hygiene practices, ventilation strategies, and occupancy limitations (33, 34). Specifically, for mechanical ventilation systems, it was imperative that the intake of air occurs exclusively from the outside and with no allowance for air recirculation. The air conditioning component of the heating, ventilation, and air conditioning (HVAC) system must remain deactivated at all times, even during group activity classes within studios. Adequate ventilation, when achieved through mechanical systems, was ensured by a six air exchanges per hour, as per the guidelines outlined (33). The number of occupants in different functional spaces of HC was strictly limited and controlled. The maximum occupants’ capacity was reduced to guarantee the physical distance of users, with a minimum of 3 m between the subjects. Strict adherence to equipment and space disinfection protocols was mandatory, with a comprehensive disinfection routine required both before and after each use of equipment or space. This responsibility rested upon both users and staff, ensuring continuous disinfection practices; the selection of the disinfection products was dictated by the respective equipment/space surface, in compliance with the prescribed guidelines (34). In addition, regular hand disinfection and other general hygiene recommendations for occupants utilizing the club facilities was emphasized (34). All HCs were equipped with HVAC systems, which at the period of the study were only used to provide a ventilation (in a limited manner Supplementary Table S1).

### 2.2 Indoor air monitoring

IAQ monitoring was conducted continuously (during 24 h) for a period of 12 days in each club, considering different indoor functional spaces. Gaseous pollutants (TVOCs, O_3_, and CO_2_) and comfort parameters (temperature – T, and relative humidity – RH) were sampled by a multi-parametric probe GrayWolf Sensing Solutions (model TG 502; GrayWolf Sensing Solutions, Shelton, USA; accuracy ±2% for TVOCs; ±3% for CO_2_ and O_3_). Particulate matter was continuously monitored by Lighthouse Handheld particle counter (model 3,016 IAQ; Lighthouse Worldwide Solutions, Fremont, USA), which allowed concurrent monitoring of six different size fractions (N_300 nm –10μm_). Particle number concentration, in a diameter range between 20 and 1,000 nm (PNC_20-1000 nm_) was monitored with TSI P-Trak™ condensation particle counter sampler, model UP 8,525 (TSI Inc., MN, USA). All pollutants were registered with logging interval of 60 s, resulting in a large set of measurements (*n* = 1,658,880). Further details on study protocol are presented in Supplementary Text S1.

### 2.3 Statistical analysis

All data were analyzed using descriptive and inferential statistical analysis using Microsoft Excel (Microsoft 365 MSO) and Statistical Package for the Social Sciences (IBM SPSS Statistics, version 28). Samples of the study population were independent and the normality of the data was verified using the Shapiro–Wilk (*n* < 30) and Kolmogorov–Smirnov (*n* > 30) tests. One sample *t* test was used for comparison with the current protective thresholds; independent samples *t* test was used to verify the influence of ventilations. The relationships between the pollutants were analyse by Spearman’s correlation coefficients. Finally linear regression was employed to model and assess the variables potentially affecting indoor air quality. All analysis were conducted using the level of statistical significance *p* < 0.05.

## 3 Results and discussion

### 3.1 Particulate matter

**Figure 1:**
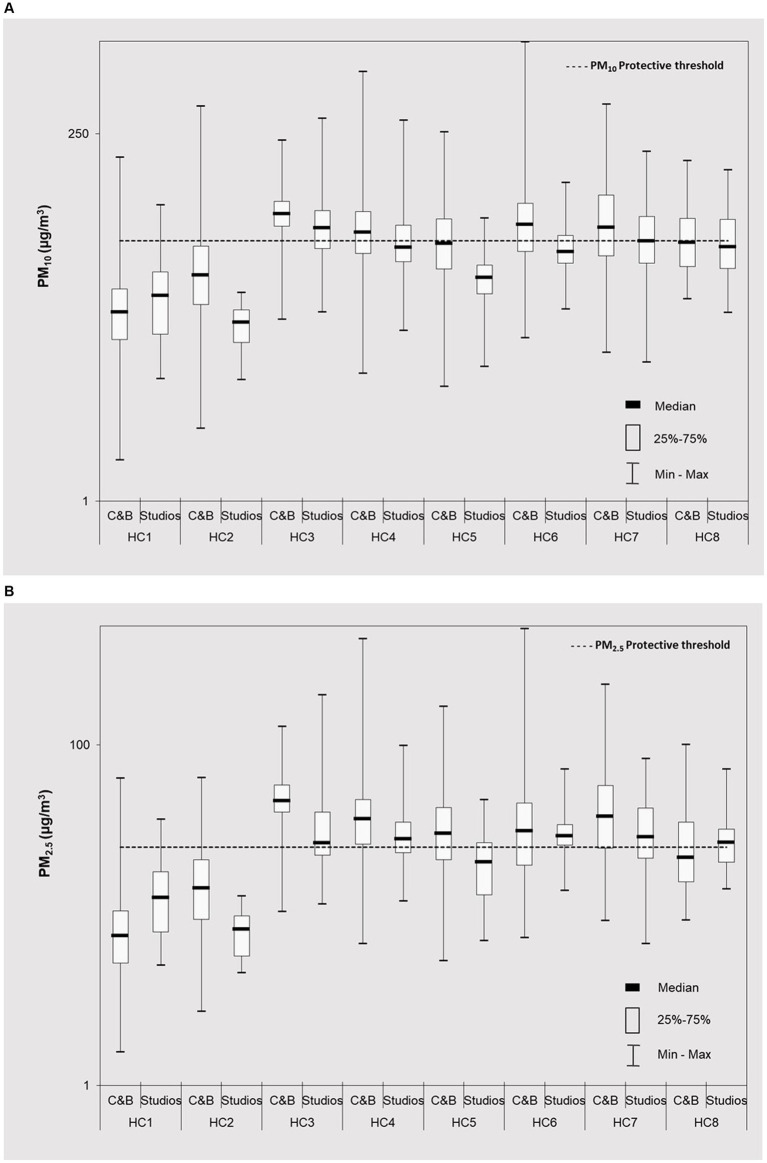

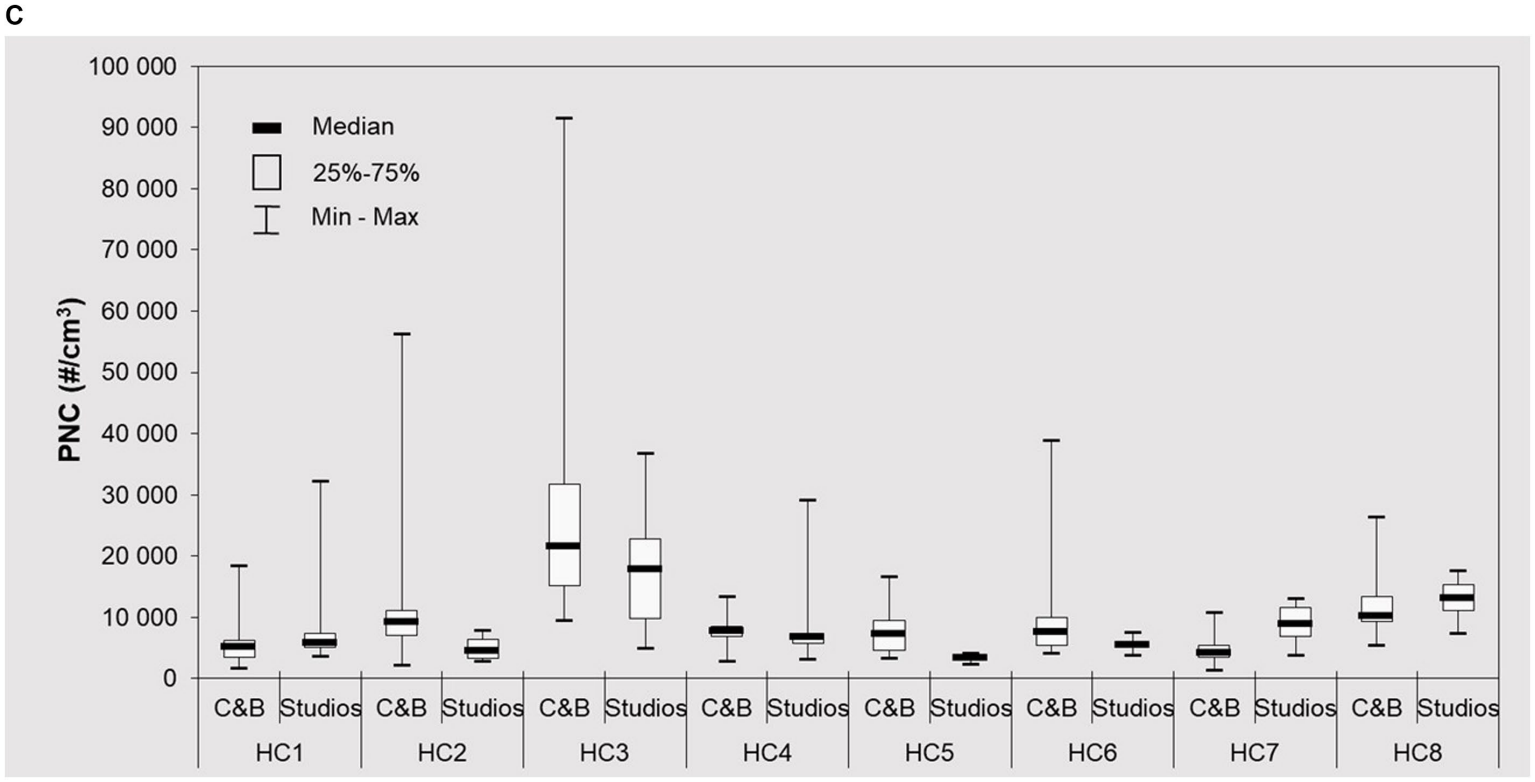
Particulate matter levels (■ median; □ 25–75%, and range) (continuous 24 h measurements) at cardio fitness & bodybuilding areas (C&B) and groups classes studios (S) and of eight health clubs (HC1– HC8) during occupied periods: **(A)** PM_10_; **(B)** PM_2.5_; and **(C)** PNC. Distributions and medians of all pollutants were significantly different (*p* < 0.05) across eight clubs and across different places. Horizontal continuous lines represent Portuguese protective thresholds for PM_10_ (50 μg/ m^3^) and PM_2.5_ (25 μg/m^3^) (35).

PM_10_ and PM_2.5_ levels according to the occupancy periods (i.e., during the opened hours and when closed) are presented in Supplementary Tables S2, S3, whereas Figures 1A,B provide graphical overviews for PM_10_ and PM_2.5_ in different functional areas across the eight HCs (when occupied). The overall results showed large variations of PM levels between the HCs and the respective functional spaces. Notably, across the eight clubs, PM_10_ medians (of each HC) ranged between 8.6–48.8 μg/m^3^ (overall median of 31.2 μg/m^3^ while PM_2.5_ was 5.8–35.9 μg/m^3^, with overall median of 21.4 μg/m^3^).

When occupied (Figure 1), the highest PM_10_ (74.9 μg/m^3^, range 15.3–225.9 μg/m^3^) and PM_2.5_ (46.9 μg/m^3^, range 10.4–127.4 μg/m^3^) were identified in HC3, being followed by HC7 (PM_10_: 60.7 μg/m^3^, range 9.3–388.1 μg/m^3^; PM_2.5_: 37.8 μg/m^3^, range 9.3–225.5 μg/m^3^).

**Figure 2:**
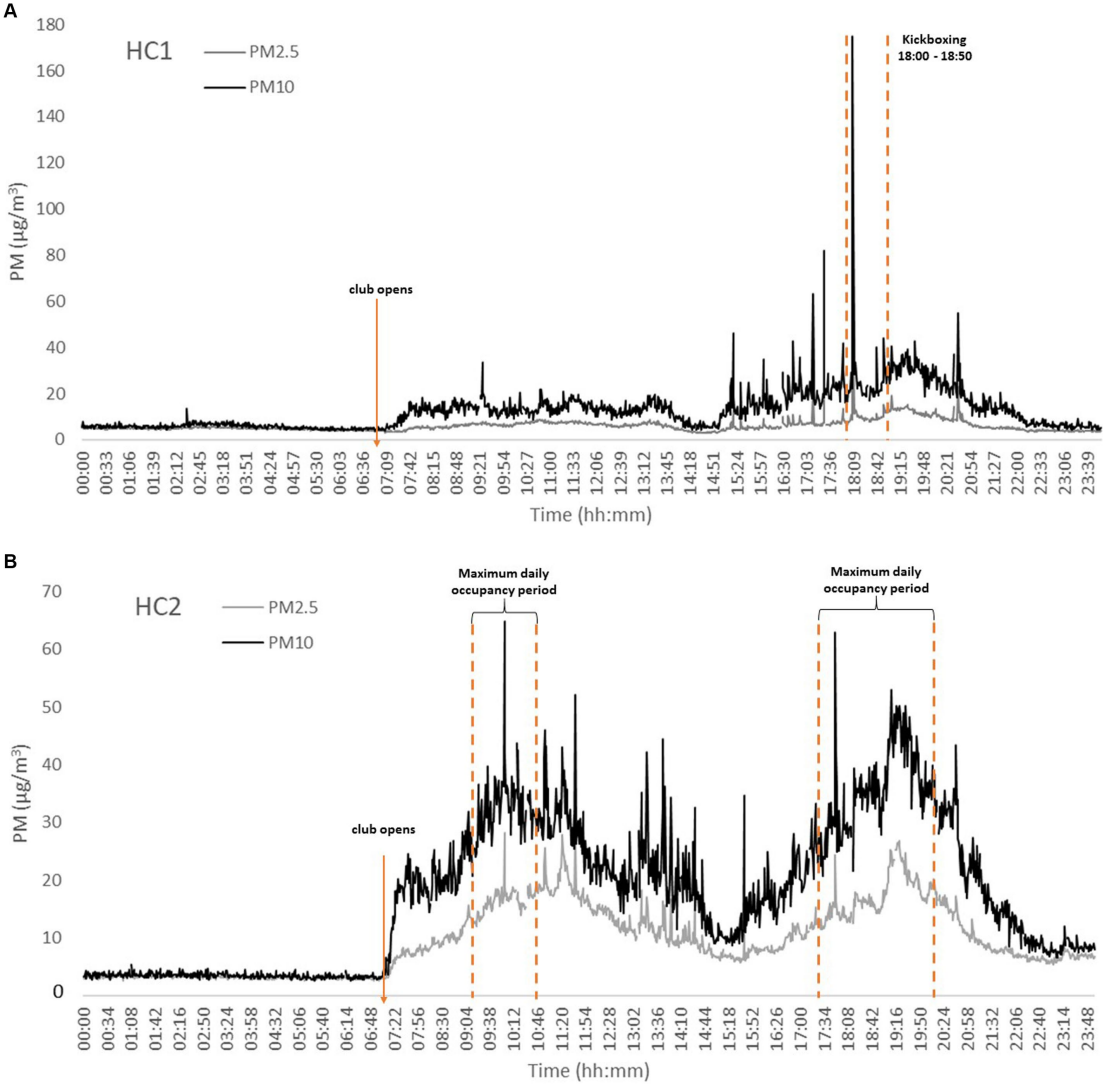

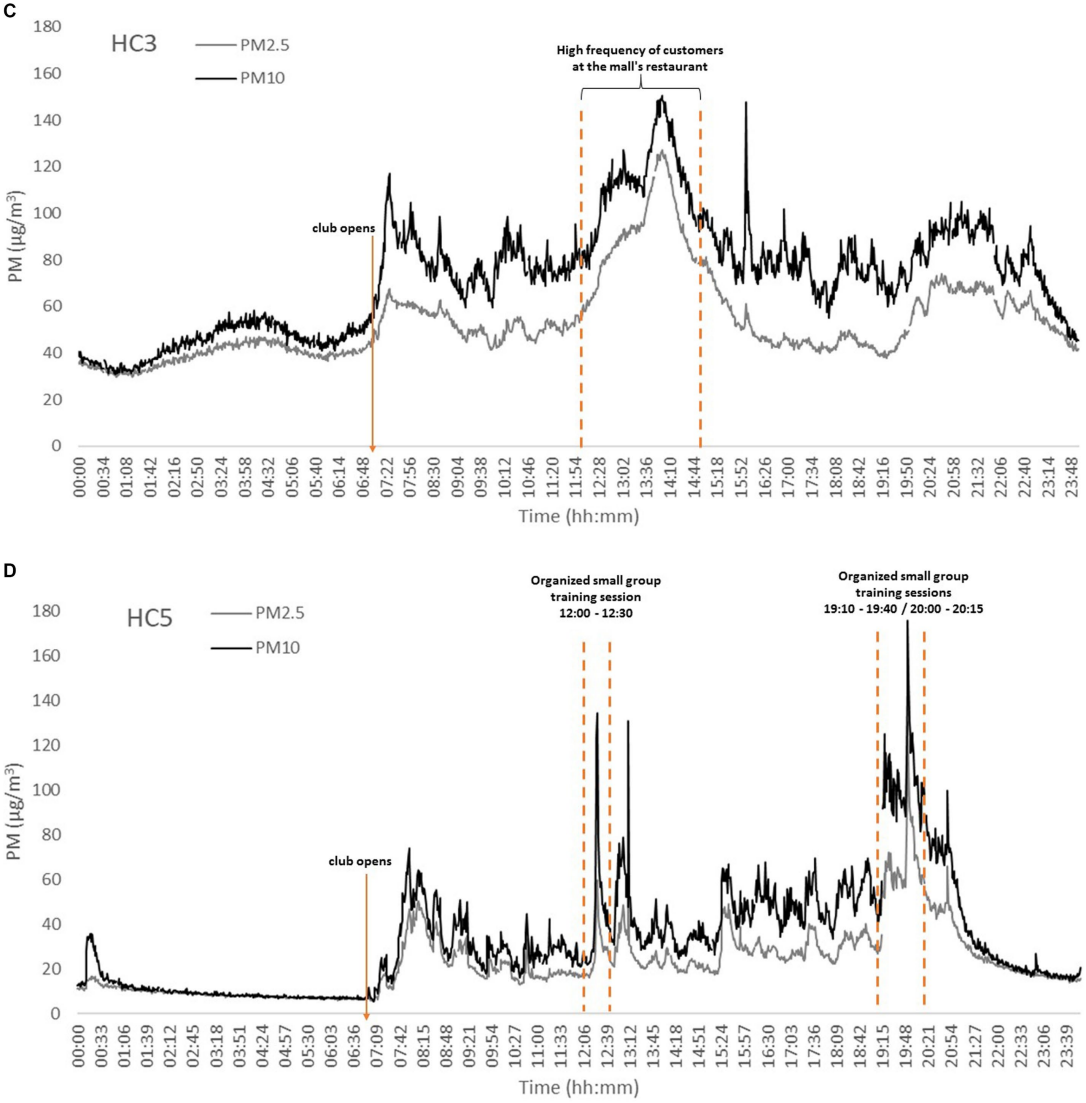

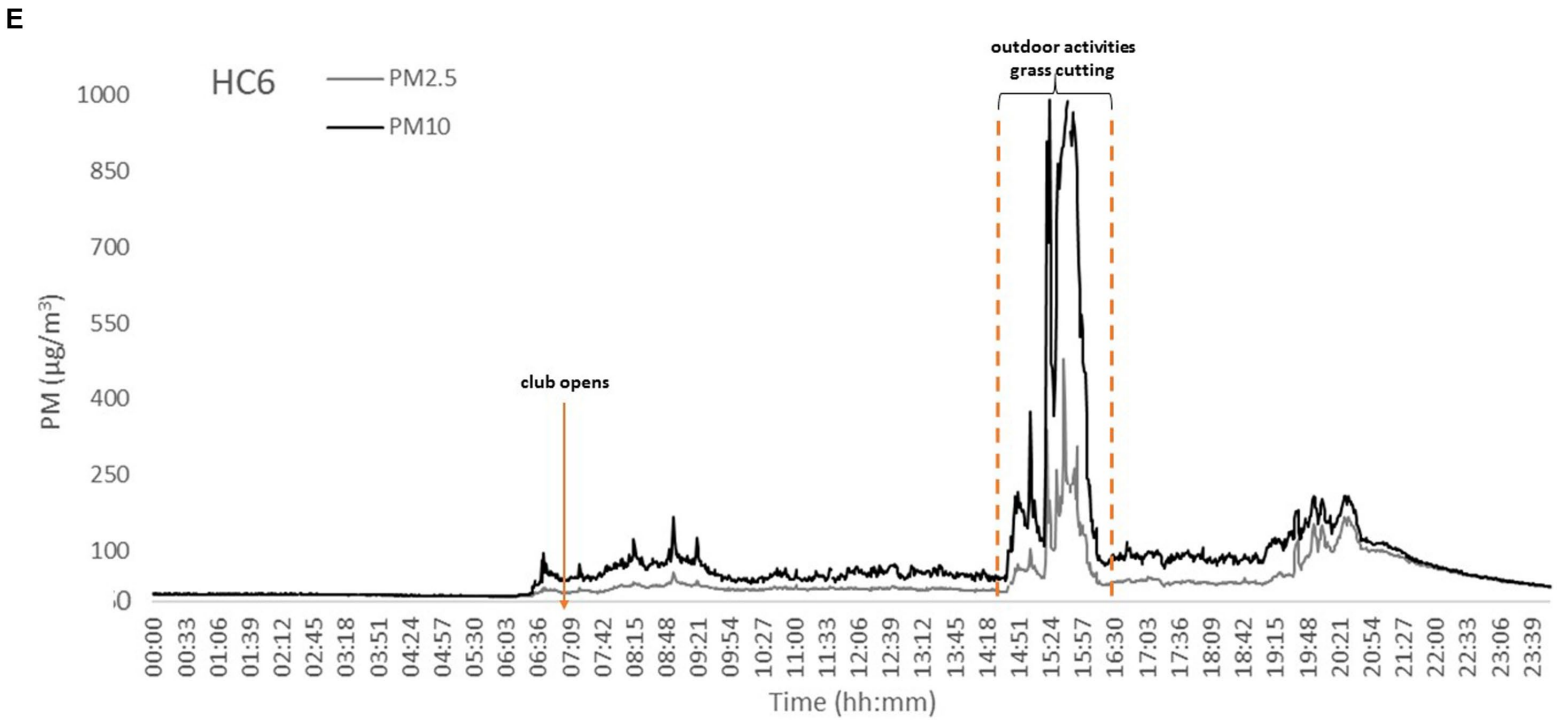
Representative PM_10_ and PM_2.5_ daily temporal variations at cardio fitness & bodybuilding areas (C&B) of the studied health clubs (HCs). PM concentrations increased when each HCs opened. **(A)** HC1 – daily profile, with a kickboxing group training; **(B)** HC2 – indication of period of greatest occupancy influx; **(C)** HC3 – high frequency of customers in surrounding restaurants; **(D)** HC5 – organized small groups training sessions; **(E)** HC6 – impact of natural ventilation.

In general, the results showed that the observed PM levels were 1.1–4.7 and 1.1–2.7 times higher for PM_10_ and PM_2.5_, respectively, when occupied (Figures 1A,B; Supplementary Table S2) than when vacant (Supplementary Table S3). These findings underline the significant influence of individual impacts and their activities on indoor PM levels (Figure 2). Consistent with the earlier studies, the observed differences were greater for coarse PM, most likely attributed to its associations with human-related sources (23, 26–28, 36). However, because of additional airing through opened windows in HC6 and HC8 (and the respective surroundings, Supplementary Table S1), it is likely that indoor PM patterns in these two clubs also resulted from outdoor emissions infiltrations. Hence, source identifications through PM mass chemical characterization would be crucial to provide further clarifications of these findings in the future investigations.

The PM obtained within this work were in similar ranges to those previously reported in studies for Lisbon (Portugal) (12, 37) or Oporto (26, 38) and in Warsaw (Poland) (24, 25). While there is plethora of data on PM from studies in educational gyms or specific sport facilities (Supplementary Table S4), the information on health and fitness clubs is sparser (Supplementary Table S4). Evaluating PM concentrations in different functional areas of the clubs showed that, in general the higher levels (up to 2.0 and 1.8 times higher for PM_10_ and PM_2.5_, respectively) were observed in C&B than in studios (Figures 1A,B). Though the C&B areas were 2–17 times higher than those of the respective studios (Supplementary Table S1), and as such allowing large particle dispersions [i.e., lower PM concentrations; (26)], the individual training is usually the preferable sport activity and thus C&B the more occupied zones used. The requirements for indoor air quality in Portuguese public buildings is established under (35) (with thresholds set as 50 μg/m^3^ and 25 μg/m^3^ for PM_10_ and PM_2.5_) (expressed as 8 h means) (Supplementary Table S5). The results showed that the average PM_10_ and PM_2.5_ was 55.3 μg/m^3^ and 32.3 μg/m^3^, respectively, exceeding than protective threshold [CI 95%, PM_10_: (48.0–62.6 μg/m^3^); PM_2.5_: (27.4–37.2 μg/m^3^)]. These results indicated the possibly health risks for the respective occupants. It is noteworthy that in studios the threshold was exceeded only in HC3 (median: 60.7 μg/m^3^), most likely due to the overall high PM levels (Figures 1A,B). Concerning PM_2.5_, the medians surpassed 25 μg/m^3^ in 63% of the HCs, in both C&B areas thus further emphasizing the potential for adverse outcomes.

As demonstrated in Figure 1, in C&B the observed temporal PM_10_ maxima reached high values, up to 989 μg/m^3^ whereas it was up to 479 μg/m^3^ for PM_2.5_. In agreement with the previous studies (Supplementary Table S4) (26), the highest temporal maxima of both PM were observed in naturally ventilated health clubs (HC6; directly facing the busy streets), due to ambient air PM indoor infiltrations (39, 40). Occasionally, grass cutting activities were recorded in the greens spaces directly outside HC6 resulting in registered extremes (Figure 2E). For the group class studios, the variations were not so high with PM_10_ concentrations between 6.2–312.4 μg/m^3^ and PM_2.5_ between 4.6–196.2 μg/m^3^.

Finally, the observed results showed that both PM_10_ and PM_2.5_ were positively and significantly correlated in all functional indoor spaces, with high Spearman correlation coefficients (r_s_) 0.830–0.961 (median of 0.909) and 0.461–0.916 (median 0.888) respectively for C&B and studios (Supplementary Table S6). Furthermore, the strong associations indicate similar origin of both PM. PM_10_ and PM_2.5_ shown a similar daily profile (Figure 2), with elevated concentrations due occupants’ physical activities (both the number of occupants and PM resuspensions caused by human movements) (8, 9, 12, 14, 26). Exercising in areas with increased PM may lead to adverse health effects, as particle deposition doubles with exercise increased intensity (41). Moreover, PM deposition into respiratory tract may be up to five times higher during moderate activity than at the rest (42). It is therefore important that reasonable measures are adopted to regulate indoor PM in order to ensure safe indoor environments that would allow for healthy exercise (43, 44).

### 3.2 Particle number concentration

Particle number concentrations in all spaces of the eight HCs (Figure 1C) showed higher levels (1.2–2.0 times) during the occupied periods than in the non-occupied one in all HCs, thus indicating the influence of occupations activity to PNC (Supplementary Figure S2C). In the C&B areas, PNC medians ranged between 4.21 × 10^3^ #/cm^3^ (HC7) and 2.15 × 10^4^ #/cm^3^ (HC3), whereas it was 3.39 × 10^3^ #/cm^3^ (HC5) and 1.78 × 10^4^ #/cm^3^ (HC3) for the group class studios. These results showed that similar concentrations range in all HCs (10^3^), with the exception of HC3 (C&B: 2.15 × 10^4^ #/cm^3^ and studios: 1.78 × 10^4^ #/cm^3^) and HC8 (C&B: 1.01 × 10^4^ #/cm^3^ and studios: 1.31 × 10^4^ #/cm^3^) where higher values were found (10^4^). Overall, the highest PNC (C&B: 2.15 × 10^4^ #/cm^3^; studios: 1.78 × 10^4^ #/cm^3^) were obtained in both functional spaces of HC3. The club unusual layout and direct connection with restaurant areas and the possible emissions (Supplementary Table S1) resulted in overall high PNC while at HC8, particle infiltrations due to natural ventilations were most likely responsible for increased PNC (26, 45–47). These results are in agreement with the previous findings that showed that mechanical ventilation systems can reduce the infiltration of ambient particles indoors (45, 46, 48). Nevertheless, no significant difference was found between HCs with mechanical ventilation vs. natural ventilation (*p* = 0.948). In addition, it shall be noted that higher PNC were observed in C&B areas than in studios in 62.5% of HCs (exception HC1, HC7, and HC8) in view of these spaces being predominately frequented.

The information on PNC in health clubs (Supplementary Table S4) is very limited. The only available information comes from study by Slezakova et al. (26) who previously reported PNC in similar overall ranges (0.5–88.6 × 10^3^ #/cm^3^), (Figure 1C). However, it needs to be emphasized that these authors observed much higher mean for each club (2.8–24.7 times) than in a present work, mainly because they assessed naturally ventilated sport facilities. In addition, on contrary to this study, the authors reported increased PNC (N_20-1000_) when spaces were unoccupied [4.8 × 10^3^ #/cm^3^ vs. 9.7 × 10^3^ #/cm^3^, (26)], due to the pollutant accumulation during the night periods (i.e., without mechanical ventilations). Slightly higher ranges of PNC (3.34 × 10^3^–15.1 × 10^3^ #/cm^3^, Supplementary Table S4) were also reported in climbing centers (49) or in stadium areas (50); obviously different PNC size fractions as well as study protocols, and sports facility layouts (partial ambient air opening) resulted in the observed differences. As epidemiological evidence indicates that PNC may cause more adverse health effects than larger particulate matter (due to the greater surface area, higher concentrations of toxic pollutants adsorbed per unit mass).

In all functional spaces, PNC showed low to moderate associations with both PM (Supplementary Table S7, C&B: PM_2.5_ r_s_ = 0.264, PM_10_ r_s_ = 0.305; studios: PM_2.5_ r_s_ = 0.496, PM_10_ *r* = 0.629), which is understood given the different characteristics and behaviors of both particle modes (3, 47). Nevertheless, PNC can be formed through secondary aerosols [i.e., the atmospheric chemistry between ozone and chemicals emitted by cleaning products, furniture or even the occupants themselves; (51)]. Still, no associations between PNC and TVOCs or O_3_ were observed, with low r_s_ in all functional spaces (Supplementary Table S7; |r_s_| = 0.110–0.269 for ozone; |r_s_| = 0.037–0.077 for TVOCs). Thus, it would be important to identify the individual VOCs in these spaces to better determine the respective associations.

### 3.3 Gaseous pollutants – TVOCs, CO_2_, and O_3_

**Figure 3:**
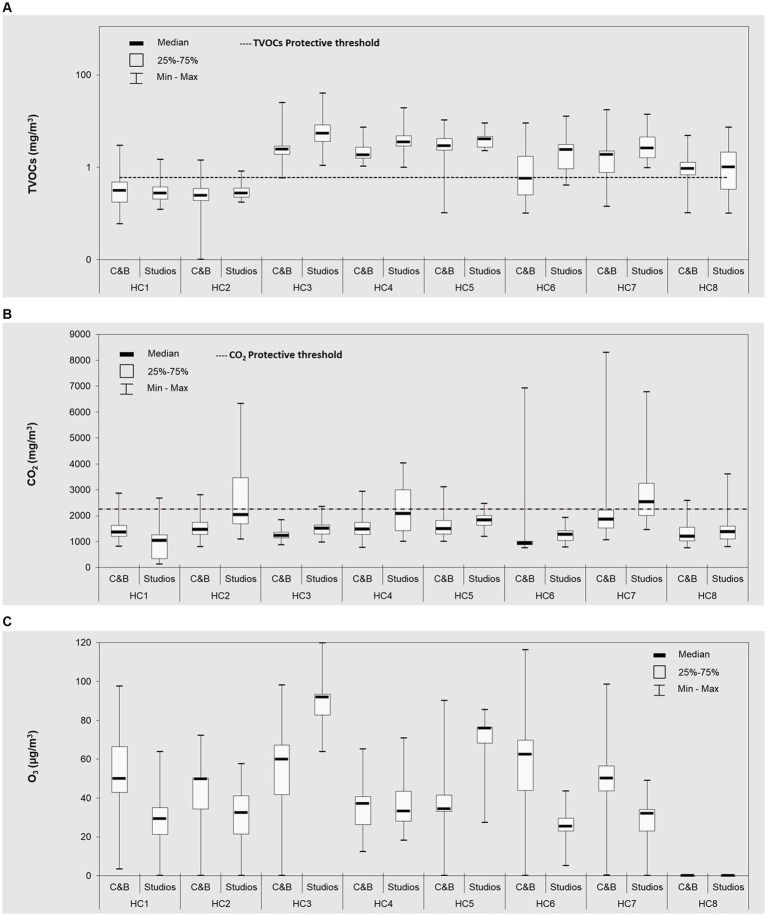
Gaseous pollutants levels (■ median; □ 25–75%, and range) (continuous 24 h measurements) at cardio fitness & bodybuilding areas (C&B) and groups classes studios (S) of eight health clubs (HC1– HC8) during the occupied periods: **(A)** TVOCs; **(B)** CO_2_ and **(C)** O_3_. Distributions and medians of all pollutants were significantly different (*p* < 0.05) across eight clubs and across different places. C&B = cardio fitness & bodybuilding areas; studios = indoor classrooms for groups activities. The horizontal lines represent the protective thresholds for TVOCs (0.6 mg/m^3^) and CO_2_ (2,250 mg/m^3^), respectively (35). For better visualization, the TVOCs graph is presented with a logarithmic scale.

Overall, the results in Figure 3 show that, the levels of TVOCs exhibited significant variations across 8 HCs (Supplementary Table S8, S9). Particularly, the concentration ranges were quite large, with medians from 0.27 (in HC2) to 3.80 (in HC3) mg/m^3^ (with an overall median of 2.34 mg/m^3^).

Considering different indoor functional spaces, the medians obtained in C&B areas (when occupied) varied between 0.24 mg/m^3^ in HC2 and 2.86 mg/m^3^ in HC5, while in group class studios the observed concentration varied between 0.27 mg/m^3^ in (HC1 and HC2) and 5.34 mg/m^3^ (in HC3). These results showed that on contrary to PM, in 88% of HCs TVOCs concentrations were 1.1–4.2 times higher in group studios that in C&B areas. This was most likely due to the specific ventilation conditions, imposed during the evaluated periods. Furthermore, the group studios volumes (Supplementary Table S1) were much smaller (∼ 1.5–17.0 times) than the respective C&B areas, thus allowing for a greater accumulation of gas compounds.

The observed TVOCs highly (0.93–5.34 μg/m^3^) exceeded the protective threshold of 600 μg/m^3^ (Supplementary Table S5) in 69% of all indoor spaces, even when considering the more restrictive median values, thus showing the high risks (3, 19). The main sources of VOC emissions in health clubs and sports centers are associated with cleaning products used to clean and disinfect space, personal hygiene products (such as perfumes, deodorants and hair products) (18, 19, 52). Some materials, namely gym equipment, such as foam padding adhesives, and flooring and carpeting are also associated with VOC emissions (18, 52). In addition, building materials (as paints, adhesives and sealants) over time emit VOCs into indoor air (19, 52). Air fresheners and scented products, as well as the use of candles and incense that were used in group classes (such as yoga/pilates) can also contribute to elevated indoor VOCs (18, 19). Still, inadequate ventilation can lead to the accumulation of VOCs indoors and/or pollutants (3, 18, 19, 53, 54). Furthermore, VOCs can also be emitted directly by humans [through exhaled breath and perspiration; (15, 16, 55, 56)] or through secondary oxidation reactions between human skin lipids and ozone (57–62). The observed results showed that in 25% of the functional spaces the median TVOCs were (1.2–2.7 times) higher (Supplementary Tables S8, S9) when occupied. However, in the majority (75%) of the all spaces (both C&B and studios), the median TVOCs were higher when unoccupied (1.1–2.0 times), most likely due to limited ventilation and accumulation of emissions (cleaning products emissions, etc.). It is though alarming that TVOCs exceeded (up to 7.3 times) the protective threshold, even during off-hours (i.e., when unoccupied). Furthermore, for naturally ventilated spaces (HC6, HC8; Supplementary Table S1), the secondary stipulations of 100% margin of exceedance were also surpassed (1.6–4.0 times) in indoor functional spaces (with the exception of the C&B area in HC6).

While there is very limited information on TVOCs in fitness clubs worldwide (Supplementary Table S10), in general, it needs to be emphasized that TVOCs observed across 8 HCs were 1.6–27.7 times higher than those reported in the previous works (12, 21, 27). During the study period, the implemented hygiene and public health regulations, required regular cleanings and sanitations processes, thus promoting the frequent use of cleaning products and disinfectants, which consequently may have led to higher TVOC pollution in the present study (33). Emphasizing the form of ventilation, Slezakova et al. (38) reported TVOCs in HC with natural ventilation (0.002–21.8 mg/m^3^) and with mechanical ventilation (0.003–12.4 mg/m^3^). In this study, the lowest TVOCs were observed in HCs with direct connections to the outside (HC1, HC2) and / or in clubs with natural ventilations (HC6 and HC8): HC2 < HC1 < HC8 < HC6. These results are in agreement with Canha et al. (63) who reported the relevance of door/ windows openings to TVOCs indoor reductions.

Overall CO_2_ median concentration across 8 HCs was 1,298 mg/m^3^, with median concentrations between 986 mg/m^3^ (HC6) – 2,040 mg/m^3^ (HC7). In all HCs, CO_2_ levels were significantly higher (up to 1.5 times) when occupied; these differences were similar in clubs with natural ventilations and mechanical ventilation (21.7–42.1% in HC6 and HC8 vs. 11.5–50.5% in other HCs). Similar to TVOCs, the lowest CO_2_ concentrations (HC6) were observed in naturally ventilated spaces (Supplementary Table S1).

**Figure 4:**
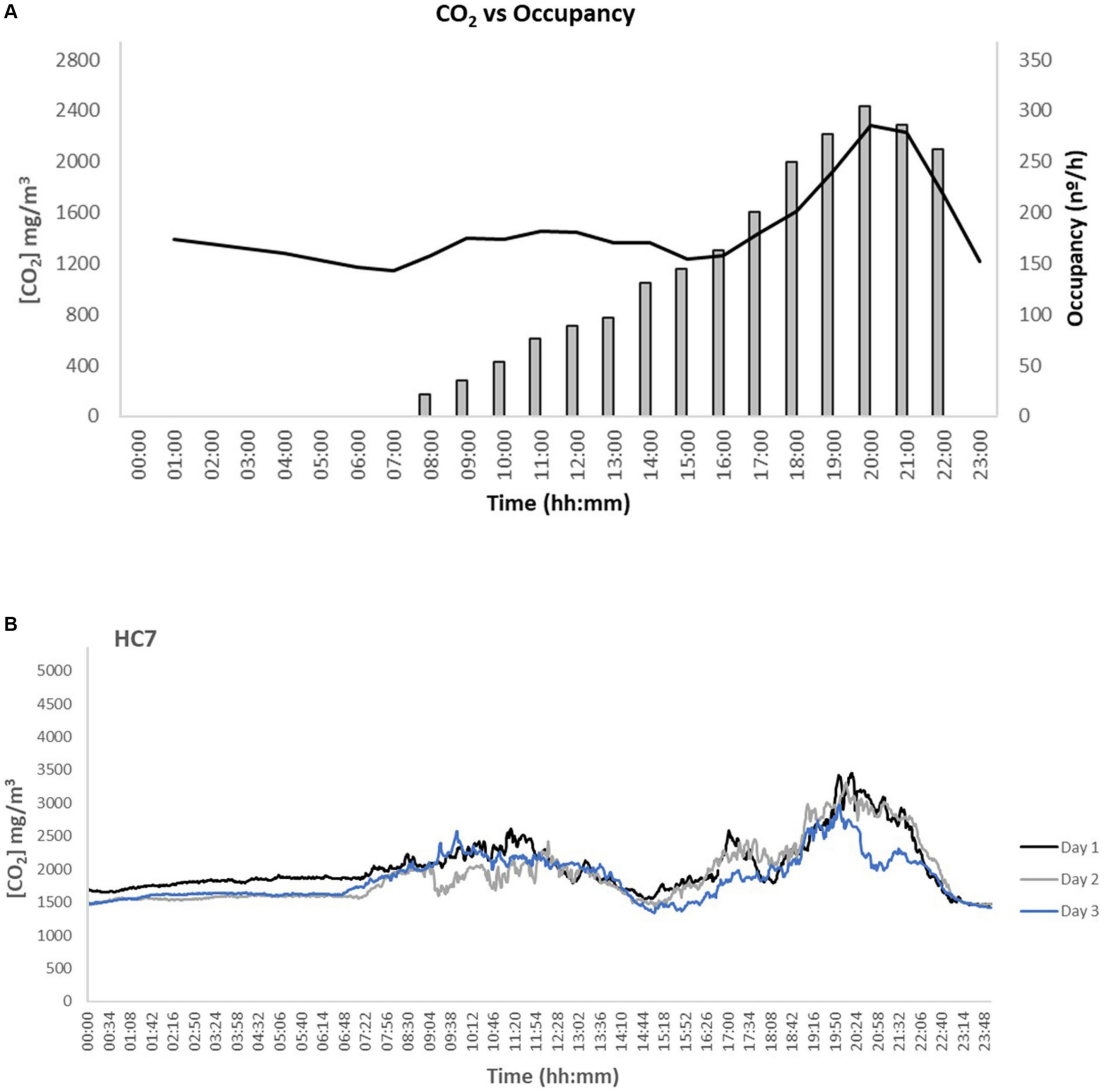
Examples of CO_2_ daily temporal variations at the studied health clubs (HCs) **(A)** daily variations of CO_2_ at HC2 and the respective occupancy; **(B)** example of continuous evolution (3 weekdays) at main workout areas of HC7 (gray scale indicates unoccupied periods).

The results showed that the average concentration of the CO_2_ was 1,489 mg/m^3^ thus fulfilling the protective threshold [CI 95% (1,389–2,338 mg/m^3^)] of 2,250 mg/m^3^. The stricter recommendation of the American Society of Heating, Refrigerating, and Air-Conditioning Engineers [ASHRAE; 1,800 mg/m^3^; (64)] was exceeded in 25% of the facilities under analysis (applying the median values of each HC). Similar to TVOCs, higher CO_2_ levels (1.1–1.4 times) were observed in group class studios (i.e., smaller room areas), with medians of 1,043 mg/m^3^ in HC1 and 2,538 mg/m^3^ in HC7, than in C&B areas (936 mg/m^3^ in HC6–1,866 mg/m^3^ in HC7). Overall, the results observed in this study were lower than those previously reported for health clubs with mechanical ventilation (Supplementary Table S10). Andrade et al. (65) analyzed 3 fitness centers (Santa Catarina, Brazil) and reported 1.3 times (2,455 mg/m^3^) and 3.4 times (6,346 mg/m^3^) higher values. Similarly, Ramos et al. (12) also observed concentrations 2.4 times higher in fitness centers in Lisbon (1,069–4,418 mg/m^3^), whereas Slezakova et al. (38) assessed HC with mechanical ventilation (26.3 times higher, 252–49,007 mg/m^3^) and natural ventilation (4.4 times higher, 697–8,122 mg/m^3^). During the period in this study, the sports facilities were subject to specific rules, also in terms of occupancy (Figure 4). As a consequence of the recent pandemic, the maximum number of users was reduced to guarantee a minimum of 3 m distance between the subjects (34). These results show that restrictions of human occupancy and distancing can promote positive impacts on reducing indoor CO_2_ pollution.

Levels of O_3_ during the occupied periods of 7 HCs are summarized in Figure 3C. In particular, the overall concentration ranges varied greatly, with medians from 38.5 μg/m^3^ (in HC9) to 79.2 μg/m^3^ (in HC3) (with an overall median of 49.0 μg/m^3^). Regarding the functional spaces, the average ozone levels varied between 34.3 μg/m^3^ (in HC5) - 68.0 μg/m^3^ (in HC6) in the C&B areas and 25.5 μg/m^3^ (in HC6) - 92.4 μg/m^3^ (in HC3) in the group class studios. In indoor air, ozone results either from infiltrations of ambient air emissions (51) or from specific indoor sources such as photocopiers, printers or air purifiers (3, 66–68); the latter were not present on sites at the time of air monitoring. Information on ozone in sports facilities is truly limited (Supplementary Table S10), which hampers the comparison between the various studies (20). While concentrations 2.8–36.6 times larger than here presented were reported for indoor air of fitness clubs (most likely due to significantly different study organizations and protocols), in partially-opened sport facility authors observed O_3_ levels in similar order of magnitude as this study (i.e., median of 84.1 μg/m^3^) (50). Similar to other PM and PNC, ozone levels exhibited 1.0–1.7 times higher levels during the occupied periods. Slezakova et al. (38) estimated that in fitness clubs with natural ventilations, ozone levels (during occupied vs. unoccupied period) were approximately twice higher (65–120%) than in clubs that were equipped with mechanical systems (∼20–80%). In this study, ozone concentrations in HCs in C&B areas with natural ventilation vs. mechanical did not differ significantly (*p* = 0.113). Concerning the occupancy impact, when people were exercising (i.e., when occupied) the observed concentrations were 0.9–1.0 times higher for HC with natural ventilations and 1.0–1.7 times higher for mechanical one. It is necessary to enhance that restricted use of mechanical ventilation system might lead to considerable implications for IAQ (69). While there is no protective threshold for ozone levels indoors, and due to its known negative health impacts, it is recommended to mitigate the levels as low as possible (70).

### 3.4 Comfort parameters

**Figure 5:**
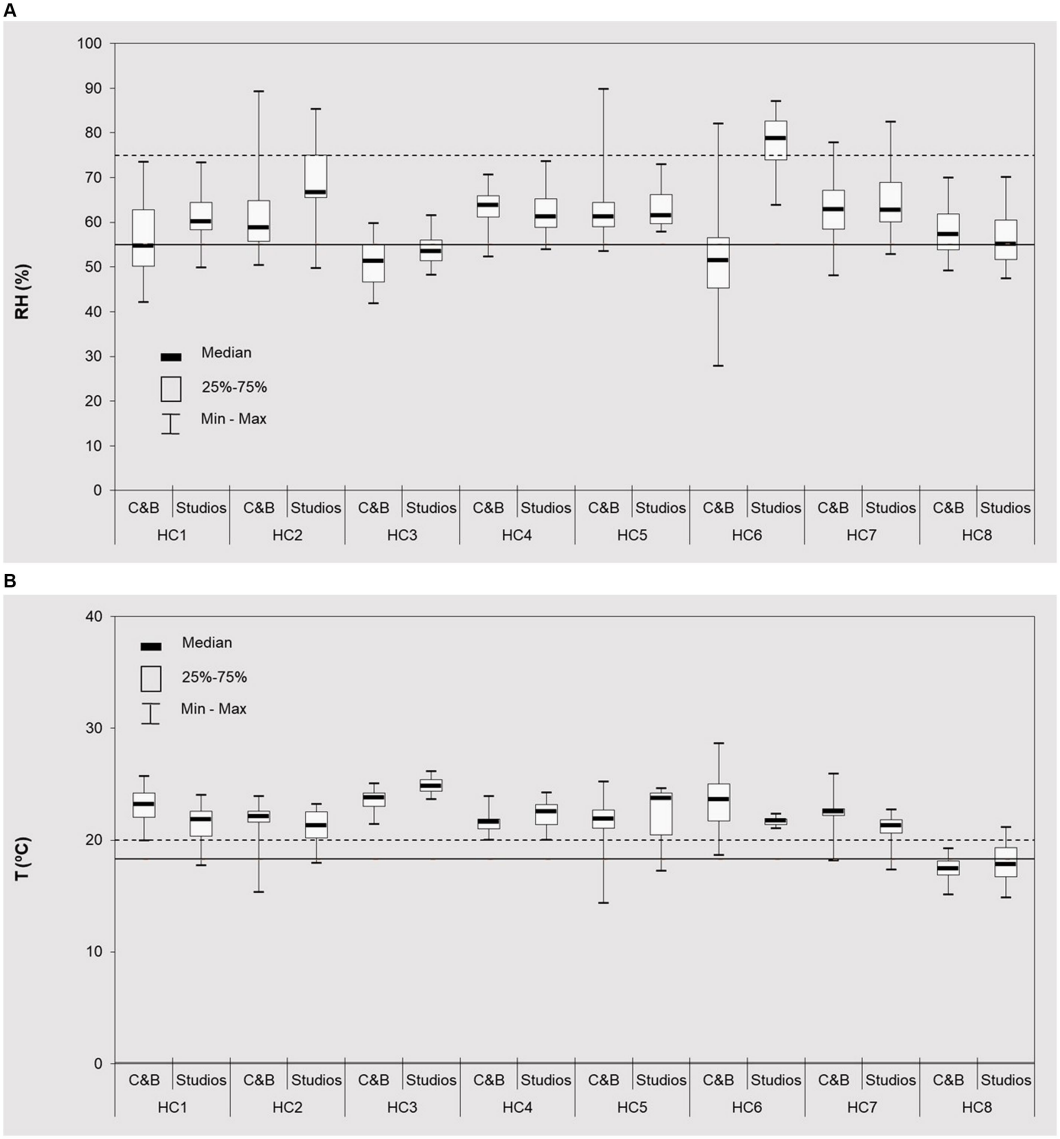
Comfort parameters levels (■ median; □ 25–75%, and range) (continuous 24 h measurements) at cardio fitness & bodybuilding areas (C&B) and groups classes studios (S) of the eight studied health clubs (HC1– HC8) during occupied periods: **(A)** relative humidity; **(B)** temperature. The horizontal lines represent guidelines for T (°C) (18.3–20°C) (73) and HR (%) (55–75%) (72).

When exercising, breathing and perspiration generate substantial amount of water vapor, which may impact the measured RH in these spaces (14). The results showed that when occupied, median RH in C&B areas varied between 51.2% (in HC3) - 63.7% (in HC4), with overall median of 57.9% (Figure 5A). Specifically for sport facilities, the guidelines for T and RH are set within the National Technical Regulation of Sports Facilities (RTID) (72), which provides the recommended values; the reference range for RH is between 55 to 75%. However, the International Fitness Association – IFA (73) considers the ideal RH in more restricted interval (40–60%). These guidelines are based on the recommendations of Occupational Safety and Health Administration - OSHA (74) and the American College of Sports Medicine – ACSM [ACSM (71)]. If taken into account the more restricted values recommended by the IFA, only 43.8% of spaces met the requirements, with the remaining values exceeding the required threshold. It should be emphasized that the limited number of studies carried out in fitness clubs under similar conditions, showed RH of the same order and magnitude as here presented [48.9–53.7%, (75); 55–65%, (23)]. However, most studies on sport facilities have shave demonstrated much wider RH ranges (Supplementary Table S10), [63.0–81.4%, (22); 49.7–99.8%, (76); 40–95%, (12)], due to high occupancies and the associated physical activities. While RH does not pose any health risks, somewhat lower values can cause certain discomfort, such drying nose, throat, mucous membranes and skin (77, 78).

Similar to TVOCs, in studios (62.5%) the observed RH were higher than in C&B, ranging from 53.5% (in HC3) - 78.7% (in HC6). In C&B areas with natural ventilation systems (HC6 and HC8), the RH was 1.1–1.2 times lower during the occupancy period than when vacant (i.e., without any form of ventilation). For all HCs with mechanical systems, the opposite trend was observed with the RH being 1.0–1.2 times higher when occupied. Hence these results emphasize the importance of ventilations and its maintenance in sport facilities when equipped with mechanical systems in order to assure optimal indoor conditions for its occupants. Furthermore, the prevention of humidity accumulation is especially relevant to have a suitable indoor microenvironment.

Regarding the temperature - T, the IFA suggests 18.3–20°C (65–68°F) for spaces where aerobics, cardio, bodybuilding and Pilates activities are carried out and 26.7°C (80°F) for yoga activities, regardless of the season. When occupied (Figure 5B), T varied between 17.4°C (in HC8) and 23.8°C (in HC3) in the C&B areas, and 17.8°C (in HC8) and 24.8°C (in HC3) in the group studios classes; no trends between both functional spaces were observed. When compared with IFA guidelines (18.3–20°C), none HC complied with the legislation guidelines. Thus, the prevention of regular air conditioning use due to the public health restrictions at the respective period (33) resulted in higher T levels, despite the lower occupancies of the sport facilities during those periods. As regular exercising in environmental conditions with T and RH changed can cause various health consequences (78–80), comfort parameters should be maintained within the recommended ranges.

## 4 Conclusion

This study assessed IAQ in different functional spaces of eight fitness centers under specific occupancy and ventilation restrictions, showing potential impacts for all pollutants and comfort parameters. Specifically, the obtained results showed that the human occupancy resulted in increased indoor particulate levels in all HCs, considering both particle number (1.2–2.0 times) and mass concentrations (1.1–4.7 times); PM_10_ and PM_2.5_ originated from the same emission sources (r_s_ of 0.888–0.909). In addition, higher levels (up 2 times for both PM and PNC) were observed in the C&B as these spaces were predominantly frequented, reinforcing the impacts of occupational activity. On the contrary, TVOCs levels were higher when HCs were unoccupied (1.1–2.0 times) emphasizing the importance of ventilation procedures to accumulation of pollutants. In addition, it needs to be emphasized that when occupied TVOCs highly exceeded the protection thresholds (1.6–8.9 times) due to the specific hygiene requirements that implied frequent use of cleaning products and disinfectants in HCs. In order to better characterize the respective health risks, identification and quantification of individual VOCs would be required. The specific rules for occupancy and human distancing in sport facilities led to positive CO_2_ impacts, with decreased levels than those previously reported for these indoor environments (12, 38, 65); the lowest CO_2_ were found in HCs with natural airing. Finally, the requirements for comfort parameters were largely (56% for RH and 100% for T) unfulfilled in all HC. These results emphasized the importance for ventilation procedures in sport facilities when equipped with mechanical systems in order to assure optimal indoor conditions for its occupants.

## Data availability statement

The original contributions presented in the study are included in the article/Supplementary material, further inquiries can be directed to the corresponding author.

## Author contributions

CP: Data curation, Formal analysis, Investigation, Writing – original draft, Writing – review & editing. MC: Funding acquisition, Supervision, Writing – review & editing. SM: Funding acquisition, Project administration, Supervision, Writing – review & editing. KS: Conceptualization, Methodology, Supervision, Writing – original draft, Writing – review & editing, Formal analysis.
