# Supplementary material for: Assessment of indoor air quality in health clubs: insights into (ultra)fine and coarse particles and gaseous pollutants
Source: Front Public Health. 2023 Nov 28;11:1310215. doi: 10.3389/fpubh.2023.1310215 (PMC10715262; doi:10.3389/fpubh.2023.1310215)
Supplement: Supplementary file 1 [file Data_Sheet_1.pdf]

Supplementary Material

Assessment of indoor air quality in health clubs: insights into (ultra)fine and coarse particles and gaseous pollutants

Cátia Peixoto[1,2], Maria do Carmo Pereira[2], Simone Morais[1], Klara Slezakova[2*]

[1]REQUIMTE–LAQV, Instituto Superior de Engenharia do Porto, Instituto Politécnico do Porto, Rua Dr. António Bernardino de Almeida 431, 4200-072 Porto, Portugal

[2]LEPABE-ALiCE, Faculdade de Engenharia da Universidade do Porto, Rua Dr. Roberto Frias, 4200-465 Porto, Portugal

Table S1 – Characterization of the eight studied health clubs (HC1 – HC8)

	HC1	HC2	HC3	HC4	HC5	HC6	HC7	HC8
Outdoor characterization	Urban traffic; Located in an industrial area with a busy-traffic main road nearby.	Urban background; With close proximity to by green zones.	Urban background; Residential environment.	Urban traffic; Area with busy vehicular traffic, next to the motorway accesses; Within proximity (500 m) to industrial zone.	Urban background; Surrounded by vegetation; Within 2 km from airport and 1 km local factories.	Urban background; Residential area with greens; 300 m distance from a national road with occasionally busy traffic.	Urban background; Area with green zones, vegetation agriculture land.	Urban traffic; City center.
Description	Located on the ground floor of a shopping center; Indoor area connected without any physical barrier.	Located sub-level of a building (car park level); Without barriers, directly connection to the road.	Located on the top floor in a shopping center; With a direct connection to the restaurant area and physical barriers to isolate the air environment of HC from the shopping mall; Unusual architectural layout; Functional zones directly connected to the restaurant areas; Ceiling fans in C&B areas.	Located in a large shopping center.	Located at building sub-level.	Located at building sub-level.	Located at street level of the buildings, direct entrance from street.	Located at ground level of the building.
Year of constructions	2018	2019	2018	2019	2019	2004	2021	1995
Building main construction materials	Brick, concrete, ceramic, polypropylene tiles, glass, plasterboard, wood and mirrors.	Brick, concrete, ceramic, polypropylene tiles, glass, plasterboard, wood and mirrors.	Brick, concrete, ceramic, polypropylene tiles, glass, plasterboard, wood and mirrors, Portuguese cobblestone (limestone).	Brick, concrete, ceramic, polypropylene tiles, glass, chipboard, plasterboard and mirrors.	Brick, concrete, ceramic, polypropylene tiles, glass, plasterboard, wood and mirrors.	Brick, concrete, ceramic, stone, glass, plasterboard, wood and mirrors.	Brick, concrete, ceramic, polypropylene tiles, glass, plasterboard, wood and mirrors.	Brick, concrete, ceramic, glass, plasterboard, wood and mirrors.
Ventilation	HVAC	HVAC	HVAC + shopping centre ventilation (The air was	HVAC	HVAC	HVAC + natural ventilation (HVAC-turned off)	HVAC	HVAC + natural ventilation (used simultaneously)

			renovated every hour)					
Occupancy (clients / day)	~ 400 - 450	~ 2300 - 2500	~ 200 - 210	~ 500 - 530	~ 300	~ 50 - 100	~ 400 - 500	~ 300 - 350
Peak occupancy	18:30 - 21:30	18:00 - 21:30	11:30 - 13:00<br>19:00 - 21:30	12:00 - 13:30<br>18:30 - 22:00	10:30 - 12:30<br>19:00 - 21:30	12:30 - 13:30<br>18:30 - 20:00	19:00 - 21:30	18:30 - 21:30
Working hours	Mon-Fri:<br>07:00 – 22:30<br>Sat:<br>08:00 – 20:00<br>Sun:<br>09:00 – 13:00	Mon-Fri:<br>07:00 – 22:00<br>Sat:<br>08:00 – 20:00<br>Sun:<br>09:00 – 13:00	Mon-Fri:<br>07:00 – 22:30 a<br>Sat:<br>08:00 – 20:00<br>Sun:<br>09:00 – 14:00	Mon-Fri:<br>07:00 – 22:30<br>Sat:<br>08:00 – 20:00<br>Sun:<br>09:00 – 13:00	Mon-Fri:<br>07:00 – 21:00<br>Sat:<br>08:00 – 15:30<br>Sun:<br>09:00 – 13:00	Mon-Fri:<br>07:00 – 14:00 / 15:00 – 21:00<br>Sat:<br>08:00 – 14:00 / 16:00 – 18:30<br>Sun: Close	Mon-Fri:<br>07:00 – 22:00<br>Sat:<br>08:00 – 20:00<br>Sun:<br>09:00 – 13:00	Mon-Fri:<br>08:00 – 22:00<br>Sat:<br>09:00 – 20:00<br>Sun:<br>09:00 – 13:00
Swimming pool	No	No	No	No	No	Yes	Yes	Yes
**Cardiofitness and bodybuilding area**								
Levels	2	1	1	2	1	1	1	1
Area (m^2):	700 / 186	503	186	307 / 56	503	287	377	289
Height (m):	7.2/ 3.9	3.2	4.4	8.8 / 3.4	3.2	3.5	2.6	3.2
Volume (m^3):	5040 / 726	1610	818	2701 / 189	1610	1005	980	925
Building main construction materials	Brick, concrete, ceramic, rubber, polypropylene tiles, glass, plasterboard, wood, mirrors, artificial grass/ turf, sheet metal, wall paint.	Brick, concrete, cork, ceramic, rubber, polypropylene tiles, artificial grass/ turf, glass, plasterboard, wood and mirrors.	Brick, concrete, rubber, sheet metal, ceramic tiles, polypropylene tiles, artificial grass/ turf, wood, mirrors and Portuguese cobblestone (limestone).	Brick, concrete, ceramic, rubber, polypropylene tiles, glass, wood, plastic blinds.	Brick, concrete, cork, ceramic, rubber, polypropylene tiles, artificial grass/ turf, glass, plasterboard, wood and mirrors.	Brick, concrete, ceramic, stone, glass, mirrors, polypropylene tiles, plasterboard, wall paint.	Brick, concrete, rubber, glass, mirrors, wood, polypropylene tiles, plasterboard.	Brick, concrete, glass, mirrors, wood, polypropylene tiles, plasterboard, and wall paint.
**Studios for group classes**								
Number:	2	2	1	2	2	1	1	1
Area (m^2):	102 / 80	128 / 260	119	114/ 67	128 / 260	105	72	145
Height (m):	2.9 / 2.9	4.1 / 4.1	4.4	6.6 / 3.4	4.1 / 4.1	3.5	3.2	2.7
Volume (m^3):	296 / 232	525 / 1066	524	749 / 226	525 / 1066	368	230	392
Building main construction materials	Glass, plasterboard, wood, mirrors, wall paint.	Brick, concrete, glass, rubber, polypropylene tiles, mirrors.	Wood, glass, mirrors, sheet metal	Brick, concrete, wood, polypropylene tiles, glass, mirrors.	Brick, concrete, glass, rubber, polypropylene tiles, mirrors.	Brick, concrete, glass, mirrors, wood, plasterboard, wall paint.	Brick, concrete, glass, rubber, mirrors, wood, polypropylene tiles, plasterboard.	Brick, concrete, wood, glass, mirrors, plasterboard and wall paint.
**Studio for cycling**								
nunber:	1	1	1	1	1	n.a.	1	1
Area (m^2):	83	63	61	69	63	-	93	61
Height (m):	2.9	5.6	4.4	3.4	5.6	-	2.6	2.9
Volume (m^3):	241	353	268	235	353	-	242	177
Building main construction materials	Glass, plasterboard, wood, ceramic, wall paint.	Brick, concrete, glass, polypropylene tiles, wood.	Wood, glass, sheet metal, polypropylene tiles.	Brick, concrete, glass, black plastic tiles, wood.	Brick, concrete, glass, polypropylene tiles, wood.	-	Brick, concrete, glass, mirrors, polypropylene tiles, wood.	Brick, concrete, mirrors, wood, plasterboard and wall paint.

Note	High-traffic main road under construction during the study period; Without barriers, directly connection to the road.		Natural ventilation	Mechanical<br>+<br>Natural ventilation

n.a.- not available

Figure S1 - Visualizations of indoor spaces at health clubs (HC1−HC8) - C&B areas.

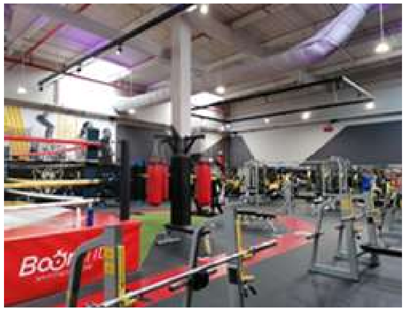

HC1

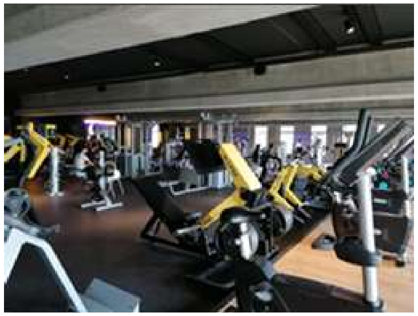

HC2

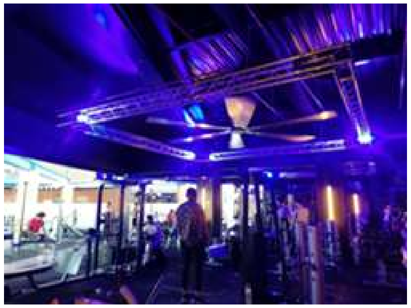

HC3

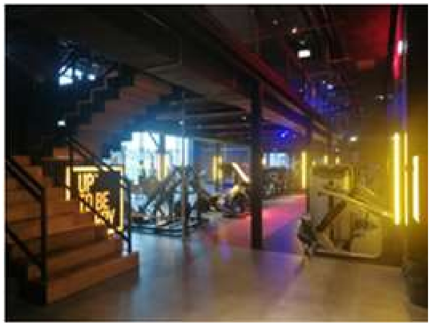

HC4

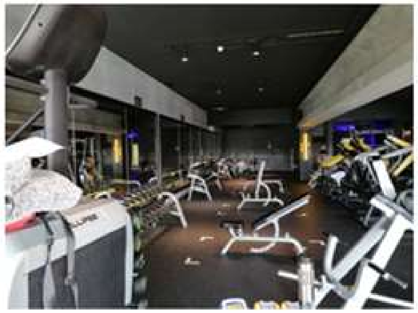

HC5

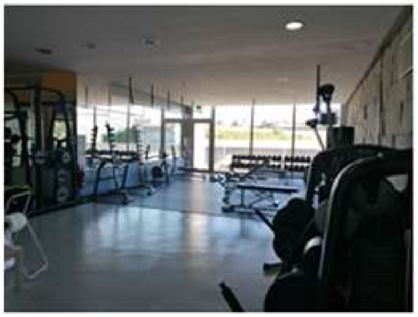

HC6

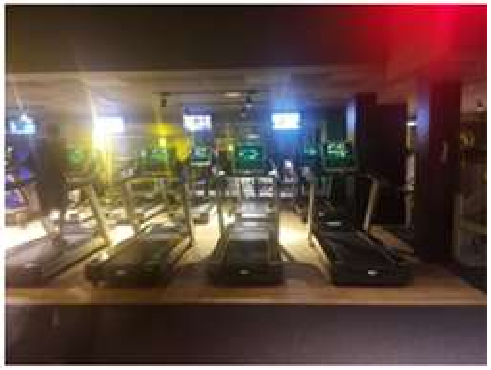

HC7

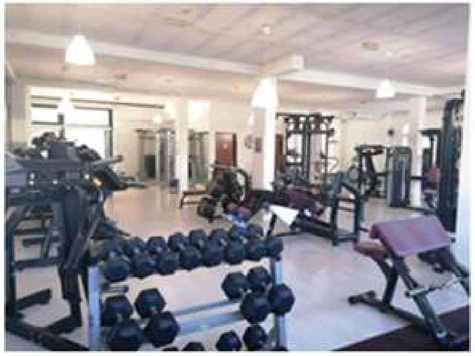

HC8

Text S1

All equipment was positioned on a support at approximately 1.5 ± 0.2 m above the floor surface and at least 1.5 m from walls to minimize the influence on particle dispersion (Holmberg and Li, 1998; Jin et al., 2013). All direct emission sources that might interfere with data acquisition (i.e., air conditioners, ventilation points, entrance exits) were avoided. Before the field campaign, the equipment was calibrated by the manufacturers. Additionally, readings of multi-gas sensor probe were weekly checked using calibration standards (difference <5%) and adjusted according to the manufacturer´s instructions. In order to minimize the occurrences of sudden artefact jumps in particle concentrations (Rivas et al., 2017), particle counter was daily zeroed (using external zeroing module). A member of the research team was consistently present at the site to record all pertinent data, including gym occupancies, class activities, and other relevant information regarding potential emission sources and ventilation conditions. Additionally, the staff of the fitness center provided supplementary details whenever necessary.

Table S2 – PM (µg/m^3) and PNC (#/cm^3) levels in indoor air of cardiofitness and bodybuilding areas (C&B) and studios (S) of eight health clubs (HC1 – HC8) during occupied periods.

		$PM_{2.5}$ (µg/m^3)		PM_{10} (µg/m^3)		PNC (#/cm^3)	
		Mean (Min - Max)	Median (25 - 75th)	Mean (Min - Max)	Median (25 - 75th)	Mean (Min - Max)	Median (25 - 75th)
HC1	C&B	8.5 (1.6 - 63.3)	7.5 (5.2 - 10.6)	19.1 (1.9 - 174.8)	17.0 (11.4 - 24.2)	5.09×10^3 (1.57 -18.2×10^3)	5.05×10^3 (3.51 – 6.28×10^3)
	S	13.7 (5.1 - 36.5)	12.6 (7.9 - 17.9)	25.1 (6.3 - 85.3)	21.9 (12.3 - 31.2)	6.83×10^3 (3.48 – 31.9×10^3)	5.84×10^3 (5.18 – 7.31×10^3)
HC2	C&B	16.1 (2.7 - 64.1)	14.3 (9.4 - 21.1)	34.5 (2.9 – 375.9)	29.8 (19.2 - 45.9)	9.76×10^3 (1.08 – 56.2×10^3)	9.16×10^3 (7.08 – 11.1×10^3)
	S	7.9 (4.6 - 12.9)	8.2 (5.7 - 9.9)	14.3 (6.2 - 22.9)	14.6 (10.8 - 17.8)	4.92×10^3 (2.63 – 7.64×10^3)	4.44×10^3 (3.28 – 6.49×10^3)
HC3	C&B	49.5 (10.4 - 127.4)	46.9 (40.2 - 58.1)	77.2 (15.3 - 225.9)	74.9 (62.2 - 90.2)	2.51×10^4 (9.32 – 91.4×10^3)	2.15×10^4 (15.3 – 31.7×10^3)
	S	34.8 (11.6 - 196.2)	26.6 (22.5 - 40.2)	65.7 (17.0 - 312.4)	60.7 (44.45 - 78.89)	1.73×10^4 (4.85 – 36.7×10^3)	1.78×10^4 (9.89 – 22.8×10^3)
HC4	C&B	42.2 (6.8 - 419.2)	36.7 (26.1 - 47.8)	66.5 (6.8 - 629.7)	56.6 (41.4 - 77.3)	7.82×10^3 (2.67 – 13.3×10^3)	7.87×10^3 (7.36 - 8.17×10^3)
	S	32.8 (12.0 - 98.8)	27.9 (23.3 - 35.2)	68.5 (12.9 - 303.3)	45.3 (36.5 - 63.1)	6.79×10^3 (2.99 – 28.9×10^3)	6.73×10^3 (6.52 – 7.53×10^3)
HC5	C&B	35.3 (5.4 - 167.5)	30.1 (21.2 - 42.9)	55.1 (5.6 - 254.6)	47.7 (32.8 - 69.7)	7.33×10^3 (3.11 – 16.5×10^3)	7.29×10^3 (4.61 – 9.46×10^3)
	S	20.2 (7.0 - 47.3)	20.4 (13.1 - 26.7)	29.6 (7.5 - 70.2)	28.6 (22.5 - 34.6)	3.34×10^3 (2.17 – 3.96×10^3)	3.39×10^3 (3.24 – 3.58×10^3)
HC6	C&B	39.1 (7.3 - 479.3)	31.1 (19.7 - 45.5)	79.7 (11.6 - 988.5)	63.7 (42.4 - 87.9)	8.22×10^3 (3.97 – 38.7×10^3)	7.60×10^3 (5.45 – 9.91×10^3)
	S	30.4 (13.9 - 71.8)	29.2 (25.7 - 33.9)	46.5 (17.8 - 119.2)	42.3 (35.7 - 54.1)	5.61×10^3 (3.67 – 7.38×10^3)	5.48×10^3 (3.51 – 5.74×10^3)
HC7	C&B	44.3 (9.3 – 225.5)	37.8 (24.8 - 57.6)	73.1 (9.3 – 388.1)	60.7 (39.9 - 99.2)	4.56×10^3 (1.23 - 10.6×10^3)	4.21×10^3 (3.51 – 5.50×10^3)
	S	33.2 (6.8 - 82.4)	28.7 (21.6 - 42.4)	55.9 (8.1 - 189.8)	49.5 (35.8 - 72.2)	8.89×10^3 (3.61 – 12.8×10^3)	8.79×10^3 (6.84 – 11.6×10^3)
HC8	C&B	28.2 (9.3 – 99.7)	21.7 (15.7 - 35.2)	56.7 (20.8 – 165.2)	48.7 (33.9 - 70.2)	1.21×10^4 (5.21 – 26.2×10^3)	1.01×10^4 (9.27 – 13.4×10^3)
	S	27.8 (14.2 - 71.5)	26.7 (20.4 - 31.9)	53.8 (16.9 - 144.5)	45.4 (33.1 - 68.9)	1.28×10^4 (7.23 – 17.5×10^3)	1.31×10^4 (11.2 – 15.4×10^3)

Table S3 – PM (μg/m^3) and PNC (#/cm^3) levels in indoor air of cardiofitness and bodybuilding areas (C&B) and studios (S) of eight health clubs (HC1 – HC8) during non-occupied periods.

		$PM_{2.5}$ (μg/m^3)		PM_{10} (μg/m^3)		UFP (#/cm^3)	
		Mean (Min - Max)	Median (25 - 75th)	Mean (Min - Max)	Median (25 - 75th)	Mean (Min - Max)	Median (25 - 75th)
HC1	C&B	4.2 (1.3 - 14.1)	4.2 (3.5 - 4.7)	5.5 (1.4 - 35.5)	5.9 (4.4 - 6.3)	4.47×10^3 (1.82 – 9.59×10^3)	4.08×10^3 (3.73 – 5.46×10^3)
	S	9.9 (4.1 - 34.6)	10.1 (9.8 - 10.3)	15.1 (4.9 - 89.6)	14.9 (13.3 - 16.8)	9.20×10^3 (1.59 – 30.9×10^3)	6.42×10^3 (5.93 – 9.68×10^3)
HC2	C&B	6.3 (2.6 - 15.1)	6.2 (5.3 - 7.0)	7.4 (2.6 - 30.1)	7.2 (6.3 - 8.1)	6.19×10^3 (1.01 – 22.1×10^3)	5.52×10^3 (3.50 – 6.85×10^3)
	S	6.4 (3.8 - 11.4)	6.4 (5.8 - 7.1)	10.4 (4.0 - 43.5)	10.4 (8.7 - 12.2)	5.00×10^3 (2.45 – 13.3×10^3)	4.81×10^3 (4.48 – 5.33×10^3)
HC3	C&B	33.9 (10.3 - 66.8)	36.0 (32.7 - 40.6)	42.7 (13.3 - 130.4)	45.2 (40.2 - 47.8)	14.0×10^3 (3.91 – 62.6×10^3)	12.1×10^3 (10.3 – 13.5×10^3)
	S	32.0 (11.0 - 75.0)	27.7 (23.7 - 39.6)	47.2 (16.7 - 140.3)	43.9 (37.8 - 57.1)	21.3×10^3 (5.42 – 59.4×10^3)	22.5×10^3 (18.5 – 22.8×10^3)
HC4	C&B	20.9 (7.9 - 63.5)	19.1 (18.5 - 21.4)	24.9 (8.8 - 331.2)	22.3 (21.6 - 27.2)	5.73×10^3 (1.25 – 9.11×10^3)	4.78×10^3 (4.45 - 7.29×10^3)
	S	31.6 (10.4 - 283.9)	33.2 (26.6 - 34.8)	47.5 (10.9 - 2316.4)	52.4 (38.4 - 55.7)	7.29×10^3 (3.13 – 14.3×10^3)	7.69×10^3 (7.38 – 7.87×10^3)
HC5	C&B	13.2 (5.0 - 58.6)	13.3 (11.9 - 15.3)	14.8 (5.0 - 94.4)	15.0 (13.3 - 17.3)	4.20×10^3 (1.15 – 8.32×10^3)	3.59×10^3 (3.49 – 4.49×10^3)
	S	14.6 (6.0 - 116.4)	11.7 (9.2 - 14.2)	17.9 (6.5 - 164.4)	14.5 (11.0 - 17.8)	3.75×10^3 (1.48 – 9.33×10^3)	3.49×10^3 (3.09 – 3.72×10^3)
HC6	C&B	24.1 (7.4 - 102.2)	25.2 (21.5 - 27.9)	29.8 (7.8 - 264.4)	30.9 (27.5 - 35.8)	6.52×10^3 (2.36 – 14.5×10^3)	6.16×10^3 (5.71 – 6.53×10^3)
	S	26.7 (10.2 - 79.4)	25.5 (22.2 - 29.9)	32.2 (10.2 - 367.7)	30.9 (28.0 - 35.2)	6.81×10^3 (4.59 – 9.77×10^3)	6.26×10^3 (5.59 – 7.43×10^3)
HC7	C&B	21.1 (6.5 - 38.9)	16.9 (15.0 - 18.9)	25.0 (6.7 - 55.2)	18.2 (15.8 - 20.9)	3.26×10^3 (1.13 – 8.61×10^3)	2.73×10^3 (2.29 – 4.69×10^3)
	S	28.4 (3.6 - 174.9)	29.5 (25.8 - 29.9)	48.0 (3.9 - 368.0)	50.0 (42.9 - 52.4)	8.89×10^3 (2.72 – 18.5×10^3)	9.61×10^3 (8.25 – 10.2×10^3)
HC8	C&B	33.4 (11.2 - 112.3)	28.6 (14.0 - 48.0)	52.2 (16.0 - 159.3)	44.8 (23.9 - 73.0)	n.d.	n.d.
	S	20.1 (5.3 - 57.7)	20.1 (14.9 - 22.0)	31.2 (6.3 - 124.5)	30.9 (27.9 - 32.6)	10.2×10^3 (4.51 – 21.8×10^3)	10.4×10^3 (8.31 – 12.3×10^3)

Table S4 - Summary of existent studies on particulate matter (PM: µg/ m^3), particle number (PN: #/ cm^3) in sport venues

Year of study	Country, city	Study description	PM fraction	Levels	Reference
**Health clubs/ fitness centers**					
2020	Netherlands<br>(Eindhoven)	Conducting 2 studies:<br><br>Study 1:<br>Measurements of endogenous (saliva) and exogenous particles (PM generated during physical exercise);<br>Study carried out in a test room equipped with air conditioning and a ventilator;<br>There was no air supply or exhaust from the room;<br>3 human volunteers who performed 2 sessions of 30 minutes each;	$PM_{10-2.5}$<br>$PM_{2.5-1}$<br>$PM_{1-0.5}$<br>$PM_{0.5-0.25}$<br>$PM_{<0.25}$	Study 1:<br>Subject 1:<br>$PM_{10-2.5}$: 1.46 µg/m^3<br>$PM_{2.5-1}$: 1.27 µg/m^3<br>$PM_{1-0.5}$: 0.35 µg/m^3<br>$PM_{0.5-0.25}$: 0.12 µg/m^3<br>$PM_{<0.25}$: 0.02 µg/m^3<br>Subject 2:<br>$PM_{10-2.5}$: 3.79 µg/m^3<br>$PM_{2.5-1}$: 1.96 µg/m^3<br>$PM_{1-0.5}$: 0.38 µg/m^3<br>$PM_{0.5-0.25}$: 0.10 µg/m^3<br>$PM_{<0.25}$: 0.02 µg/m^3<br>Subject 3:<br>$PM_{10-2.5}$: 6.38 µg/m^3<br>$PM_{2.5-1}$: 2.12 µg/m^3<br>$PM_{1-0.5}$: 0.36 µg/m^3<br>$PM_{0.5-0.25}$: 0.10 µg/m^3<br>$PM_{<0.25}$: 0.01 µg/m^3	Blocken et al., 2021
2014	Portugal<br>(Oporto)	4 fitness centres with cardiofitness and bodybuilding areas and studios group classes;<br>Continuous measurements (7 days – 24h per day), during 40 days (May - June 2014);	$PM_{2.5}$<br>PM_{10}<br>UFP	$PM_{2.5}$: 5 - 777 µg/m^3 (median: 5-37 µg/m^3)<br>FC1: 5 - 285 µg/m^3 (median: 37 µg/m^3)<br>FC2: 6 - 777 µg/m^3 (median: 15 µg/m^3)<br>FC3: 11 - 76 µg/m^3 (median: 19 µg/m^3)<br>FC4: 5 - 104 µg/m^3 (median: 15 µg/m^3)<br><br>PM_{10}: 5-1080 µg/m^3 (median: 15-43 µg/m^3)<br>FC1: 5-459 µg/m^3 (median: 43 µg/m^3)<br>FC2: 6-1080 µg/m^3 (median: 22 µg/m^3)<br>FC3: 11-95 µg/m^3 (median: 22 µg/m^3)<br>FC4: 6-106 µg/m^3 (median: 15 µg/m^3)<br><br>UFP: 0.5 - 88.6x10^3 #/cm^3<br>OC: 4.8x10^3 #/cm^3<br>NOC: 9.7x10^3 #/cm^3<br>FC1: 2463 - 46543 #/cm^3<br>FC2: 916 - 28731 #/cm^3<br>FC3: 1004 - 9916 #/cm^3<br>FC4: 474 - 88600 #/cm^3	Slezakova et al., 2018 a
2014	Portugal<br>(Oporto)	4 health clubs with cardiofitness and bodybuilding areas and studios group classes;	PM_1<br>PM_4	PM_1:<br>HC1: 5 - 328 µg/m^3 (median: 36 µg /m^3)	Slezakova et al., 2018 b

		HC1, HC2: without pool;<br>HC3, HC4: with pool and HVAC system ventilation;<br>Continuous measurements (7 days – 24h per day), during 40 days (May - June 2014);		HC2: 6 - 638 μg/m^3 (median: 20 μg/m^3)<br>HC3: 11 - 75 μg/m^3 (median: 19 μg/m^3)<br>HC4: 3 - 105 μg/m^3 (median 15 μg/m^3)<br>PM_4:<br>HC1: 5 - 368 μg/m^3 (median: 38 μg/m^3)<br>HC2: 6 - 829 μg/m^3 (median: 21 μg/m^3)<br>HC3: 11 - 78 μg/m^3 (median 20 μg/m^3)<br>HC4: 3 - 102 μg/m^3 (median 14 μg/m^3)	
2014	Thailand (Nakhon Pathom Province)	3 fitness centers: 2 indoor (municipality and university complexes) and 1 outdoor open-air facility;<br>Field measurements conducted 3 times (beginning, middle and end of operating hours) over period of 39 days;	PM_{10}	PM_{10}: 51 - 62 μg/m^3 (16 - 163 μg/m^3)	Onchang and Panyakapo, 2016
2012	Portugal (Lisbon)	Measurements the concentrations of the pollutants in a monitoring program performed in 63 fitness classes (aerobic (A) and holistic (H));<br>Duration of each class: 45 min;<br>Data time resolution: 1 minute;	$PM_{0.5}$<br>PM_1<br>$PM_{2.5}$<br>PM_5<br>PM_{10}	$PM_{0.5}$<br>A: 2.9 ± 1.7 μg/m^3 (0.52 - 9.3 μg/m^3)<br>H: 2.4 ± 0.86 μg/m^3 (1.6 - 4.4 μg/m^3)<br>PM_1<br>A: 4.6 ± 2.5 μg/m^3 (0.70 - 15 μg/m^3)<br>H: 3.6 ± 1.3 μg/m^3 (2.9 - 6.9 μg/m^3)<br>$PM_{2.5}$<br>A: 8.3 ± 4.5 μg/m^3 (0.89 - 34 μg/m^3)<br>H: 5.5 ± 2.2 μg/m^3 (3.5 - 12 μg/m^3)<br>PM_5<br>A: 18 ± 10 μg/m^3 (1.4 - 89 μg/m^3)<br>H: 10 ± 6.3 μg/m^3 (4.2 - 30 μg/m^3)<br>PM_{10}<br>A: 31 ± 23 μg/m^3 (1.8 - 153 μg/m^3)<br>H: 14 ± 8.9 μg/m^3 (4.2 - 42 μg/m^3)	Ramos et al., 2015
2012	Portugal (Lisbon)	11 fitness centres with fitness/ bodybuilding area + 2 studios (during classes);<br>Short-term (45-60 min) measurements conducted in the most occupied periods (late afternoon/ night);<br>3 fitness centres with continuos measurements (approx. 6 days);<br>9 fitness centres with mechanical ventilations;	PM_1<br>$PM_{2.5}$<br>PM_{10}	PM_1: 0.9 - 16 μg/m^3 (0.74 - 18 μg/m^3)<br>$PM_{2.5}$: 1.5 - 23 μg/m^3 (0.9 - 43 μg/m^3)<br>PM_{10}: 3.5 - 101 μg/m^3 (1.8 - 153 μg/m^3)	Almeida et al., 2015; Ramos et al., 2014
2008-2010	Germany (Munich, Stuttgart, Hanau, Regensburg)	4 different indoor climbing centers;<br>Measurements on 1 evening during 7 consecutive weeks;	PM_1<br>$PM_{2.5}$<br>PM_{10}<br>PN	PM_1: 5 - 23 μg/m^3<br>$PM_{2.5}$: 19 - 160 μg/m^3<br>PM_{10}: 129 - 1179 μg/m^3<br>PN: 3335 - 15070 #/cm^3	Weinbruch et al., 2012
n.s.	Greece (Athens)	Personal exposure assessment study;<br>Various indoor spaces including 1 gym ;<br>Used TSI Sidepak portable sampling pumps (SP530);	PM_4	PM_4: 116 - 284 μg/m^3	Saraga et al., 2014

		Sampler placed at the breathing level of the volunteers (2), for approx. 8h;<br>Duration for 3 consecutive days;<br>Quantification by gravimetric method;			
**Sport facilities**					
2020	China<br>(Hangzhou)	1 badminton hall;<br>Measurements during the wet season;<br>2 types of approach: continuous measurements + surveys;	$PM_{2.5}$<br>PM_{10}	$PM_{2.5}$: 75 µg/m³<br>PM_{10}: 150 µg/m³	Xie et al., 2021
2019	Japan<br>(Yokohama)	Championship: main stadium and warm-up zone;<br>Monitoring before and during the championship (9-12 May);<br>Study protocol: UFP and $PM_{2.5}$: 1 data time resolution;<br>BC: 5 minutes; 4 gravimetric $PM_{2.5}$;<br>wind speed and direction monitoring: stadium and on the warm-up track;<br>Quantification of exposure to PAHs measured with the help of volunteers who attended the same areas as the athletes with the help of silicone wristbands, between 12:00 on May 8 and 22:00 on May 12	PM_1<br>$PM_{2.5}$<br>PM_{10}<br>UFP<br>BC	Median (IQR)<br>Stadium:<br>PM_1: 4.6 µg/m³ (3.8 - 5.9 µg/m³)<br>$PM_{2.5}$: 8.4 - 17.8 µg/m³ (6.5 - 22.4 µg/m³)<br>PM_{10}: 15.6 µg/m³ (11.8 - 22.6 µg/m³)<br>UFP: 8968 #/cm³ (6285 - 11365 #/cm³)<br>BC: 737 ngBC/m³ (400 - 1178 ngBC/m³)<br><br>Warm-up track:<br>PM_1: 3.6 µg/m³ (2.7 - 4.5 µg/m³)<br>$PM_{2.5}$: 6.9 µg/m³ (5.2 - 8.9 µg/m³)<br>PM_{10}: 14.1 µg/m³ (9.9 - 19.9 µg/m³)<br>UFP: 11124 #/cm³ (7786 - 14523 #/cm³)<br>BC: 862 ngBC/m³ (578 - 1260 ngBC/m³)<br><br>PAHs:<br>Acenaphthene: 213.43 ng/wristband<br>Acenaphtylene: 172.29 ng/wristband<br>Fluorene: 292.14 ng/wristband<br>Phenanthrene: 545 ng/wristband	Reche et al., 2020
2017	Poland<br>(Warsaw)	1 sports facility;<br>Air monitoring: 8 h/day, for 20 days in two seasons (with + without heating);<br>TSP bound-metals and PAHs;<br>Exposure of its users, 8-40 years old;	PM_1<br>$PM_{1-2.5}$<br>$PM_{2.5-4}$<br>PM_{4-10}<br>PM_{10-100}<br>TSP	Non-heating season:<br>I:<br>$PM_{1-2.5}$: 8 - 174 µg/m³<br>PM_4: 9 - 179 µg/m³<br>PM_{10} (max): 215 µg/m³<br>TSP (max): 380 µg/m³<br>O:<br>$PM_{1-2.5}$: 3 - 174 µg/m³<br>PM_4: 3 - 174 µg/m³<br>PM_{10} (max): 180 µg/m³<br>TSP (max): 190 µg/m³<br><br>Heating season:<br>PM_1, $PM_{2.5}$, PM_4 and PM_{10}<br>(Similar Non-heating season)<br>9 - 168 µg/m³	Bralewska et al., 2020

				TSP<br>I: 10 - 500 μg/m^3<br>O: 6 - 174 μg/m^3	
2017	Poland (Warsaw)	1 sports hall;<br>Measurements simultaneously inside and outside;<br>Two measurement campaigns:<br>20 consecutive working days during the non-heating period (warm season from May–June 2017);<br>20 consecutive working days in the heating season (cold season from October–November 2017);	PM_1<br>$PM_{2.5}$<br>PM_4<br>PM_{10}<br>TSP	Non-Heating Season:<br>I:<br>PM_1: 29 μg/m^3 (8 - 172 μg/m^3)<br>Median: 13 μg/m^3<br>$PM_{2.5}$: 30 μg/m^3 (8 - 174 μg/m^3)<br>Median: 13 μg/m^3<br>PM_4: 31 μg/m^3 (9 - 179 μg/m^3)<br>Median: 14 μg/m^3<br>PM_{10}: 40 μg/m^3 (11 - 217 μg/m^3)<br>Median: 19 μg/m^3<br>TSP: 62 μg/m^3 (13 - 379 μg/m^3)<br>Median: 39 μg/m^3<br>O:<br>PM_1: 22 μg/m^3 (3 - 169 μg/m^3)<br>Median: 14 μg/m^3<br>$PM_{2.5}$: 23 μg/m^3 (3 - 174 μg/m^3)<br>Median: 14 μg/m^3<br>PM_4: 24 μg/m^3 (3 - 174 μg/m^3)<br>Median: 15 μg/m^3<br>PM_{10}: 27 μg/m^3 (4 - 179 μg/m^3)<br>Median: 15 μg/m^3<br>TSP: 32 μg/m^3 (6 - 190 μg/m^3)<br>Median: 17 μg/m^3<br><br>Heating Season:<br>I:<br>PM_1: 38 μg/m^3 (9 - 142 μg/m^3)<br>Median: 16 μg/m^3<br>$PM_{2.5}$: 39 μg/m^3 (9 - 143 μg/m^3)<br>Median: 16 μg/m^3<br>PM_4: 40 μg/m^3 (9 - 146 μg/m^3)<br>Median: 16 μg/m^3<br>PM_{10}: 45 μg/m^3 (10 - 171 μg/m^3)<br>Median: 18 μg/m^3<br>TSP: 56 μg/m^3 (10 - 500 μg/m^3)<br>Median: 29 μg/m^3<br>O:<br>PM_1: 52 μg/m^3 (5 - 164 μg/m^3)<br>Median: 34 μg/m^3<br>$PM_{2.5}$: 52 μg/m^3 (5 - 164 μg/m^3)<br>Median: 34 μg/m^3<br>PM_4: 53 μg/m^3 (5 - 165 μg/m^3)<br>Median: 34 μg/m^3	Bralewska et al., 2019

				PM_{10}: 55 µg/m^3 (6 - 168 µg/m^3)<br>Median: 35 µg/m^3<br>TSP: 59 µg/m^3 (6 - 174 µg/m^3)<br>Median: 36 µg/m^3	
n.s.	Malasya (Shah Alam)	1 university gym;<br>Old (22 yrs) airtight sealed building ;<br>Measurements made using a Multiparameter Environmental Monitoring (EVM-7);	PM	PM: 61.5 µg/m^3<br>PM_{max}: 125 µg/m^3	Mohd Hashim et al., 2019
2017	Poland (Warsaw)	1 sports hall - multifunctional facility (used for different sport and trainings);<br>Study period: 15 days (December - January 2017);<br>Simultaneously inside and outside monitoring;<br>Approximate duration: 436 minutes;	TSP<br>PM_4<br>PAHs	TSP:<br>Indoor: 115.1 ± 9.6 µg/m^3<br>Outdoor: 65.2 ± 34.7 µg/m^3<br>PM_4:<br>Indoor: 74.2 ± 21.5 µg/m^3<br>Outdoor: 22.3 ± 8.2 µg/m^3	Kuskowska et al., 2018
2015	Portugal (Oporto)	Sports environments: 2 indoor + 2 outdoor places for exercising;<br>Monitoring for 4 weeks (May and June);<br>Personal exposure to UFP;	UFP	I1: 11861 #/cm^3<br>I2: 14200 #/cm^3<br>Median: 7653 #/cm^3<br><br>O1: 20367 #/cm^3<br>O2: 7122 #/cm^3<br>Median: 12563 #/cm^3	Slezakova et al., 2019 a
2013	Kazakhstan (Astana)	2 university sports facilities - gymnastics hall and multi-propose hall;<br>Personal exposure: teenagers, adults, and students;<br>Monitoring for 2 weeks (February to March, 2013);<br>Sunday considered as a reference period in view of no training sessions;	PM_1<br>$PM_{2.5}$<br>PM_4<br>PM_{10}<br>PM_{Total}	Gymnastics hall:<br>PM_1: 7.29 µg/m^3 (2.0 - 43.0 µg/m^3)<br>$PM_{2.5}$: 7.71 µg/m^3 (2.0 - 44.0 µg/m^3)<br>PM_4: 7.86 µg/m^3 (2.0 - 46.0 µg/m^3)<br>PM_{10}: 9.86 µg/m^3 (2.0 - 86.0 µg/m^3)<br>PM_{Total}: 24.14 µg/m^3 (2.0 - 243.0 µg/m^3)<br>Multi-propose hall:<br>PM_1: 7.75 µg/m^3 (3.0 - 24.0 µg/m^3)<br>$PM_{2.5}$: 8.25 µg/m^3 (3.0 - 24.0 µg/m^3)<br>PM_4: 8.25 µg/m^3 (3.0 - 24.0 µg/m^3)<br>PM_{10}: 9.25 µg/m^3 (4.0 - 26.0 µg/m^3)<br>PM_{Total}: 13.0 µg/m^3 (4.0 - 33.0 µg/m^3)	Amouei Torkmahalleh et al., 2018
n.s.	Czech Republic (Prague)	1 university sport facilities;<br>3 spaces: 2 conventional gyms, 1 fitness centre;<br>Air monitoring during different periods: empty rooms/without ventilation and during normal occupancy (with students/standard ventilation);	PM_1<br>$PM_{2.5}$<br>PM_4<br>PM_{10}	Empty rooms/without ventilation:<br>PM_1: 66 - 72 µg/m^3<br>$PM_{2.5}$: 72 - 76 µg/m^3<br>PM_4: 73 - 78 µg/m^3<br>PM_{10}: 56 - 80 µg/m^3<br>Occupied/with ventilations:<br>PM1: 20 - 22 µg/m^3<br>PM2.5: 23 - 25 µg/m^3<br>PM4: 25 ± 1 µg/m^3	Kic, 2016

				PM10: 29 - 32 µg/m^3	
2013	Korea	64 screen golf courses located in the capital area;<br>Duration: September to November 2013, without the distinction of seasons, at the game rooms (G) and lobbies (L).	PM_{10}	PM_{10}<br>G: 72.2 µg/m^3 (24.6 - 270.7 µg/m^3)<br>L: 70.6 µg/m^3 (22.5 - 289.8 µg/m^3)	Goung et al., 2014
2013	Portugal<br>(Oporto, Lisbon)	3 sports halls (for gymnastics);<br>Air monitoring during winter (3 days) and spring (2 days);<br>Measurements taken on the busiest day of the week and during opening hours;<br>Approx. 4h measurements.	$PM_{2.5}$<br>PM_{10}	Spring:<br>$PM_{2.5}$: 500 µg/m^3 (18 - 379 µg/m^3)<br>PM_{10}: 716 µg/m^3 (250 - 1270 µg/m^3)<br>Winter:<br>$PM_{2.5}$: 170 µg/m^3 (36 - 405 µg/m^3)<br>PM_{10}: 658 µg/m^3 (77 - 1200 µg/m^3)	Filipe et al., 2013
2012	Spain<br>(Leon)	1 university gymnasium;<br>1 week monitoring;<br>Occupied vs. background assessment (during weekends);<br>Emphasis on PM distribution during various physical activities;	PM_1<br>$PM_{2.5}$<br>PM_{10}<br>$PM_{>10}$<br>TSP	With various activities/situations:<br>PM_1: 3 - 14 µg/m^3<br>$PM_{2.5}$: 6 - 90 µg/m^3<br>PM_{10}: 13 - 700 µg/m^3<br>$PM_{>10}$: 2 - 100 µg/m^3<br>TSP: 13 - 800 µg/m^3<br>Background (during weekend):<br>PM_1: 2.1 µg/m^3<br>$PM_{2.5}$: 2.7 µg/m^3<br>PM_{10}: 3.2 µg/m^3<br>$PM_{>10}$: n.d.<br>TSP: 3.2 µg/m^3	Castro et al., 2015
2012	Spain<br>(Leon)	University facilities: 1 gymnasium and 1 fronton (court to play paddle ball);<br>1 week consecutive measurements;<br>Weekdays (occupied) vs. background assessment(weekends);<br>Emphasis on PM composition.	PM_{10}	Gym:<br>Weekend: 16.8 µg/m^3<br>Weekdays: 177 ± 17.4 µg/m^3<br>Fronton:<br>Weekend: 13.3 µg/m^3<br>Weekdays: 40.0 ± 3.5 µg/m^3	Alves et al., 2013, 2014
2011	Slovenia<br>(Ljubljana)	1 university gym hall;<br>1 month-long measurements (March to April 2011) (PM + comfort parameters) during sporting activities;<br>735 dust samples (every 1h) were collected by the 2D-step sampler;	PM_{10}	Single person's contribution:<br>PM_{10}: increase of 1.5 ± 0.3 µg/m^3 (per person per slot);<br>Monthly average PM_{10}: 33 µg/m^3	Zitnik et al., 2016
**Educational facilities**					
2015	Spain<br>(Barcelona)	2 school gyms naturally ventilated;<br>Period: February and April 2015;<br>4 different ventilation strategies analyzed: natural ventilation, manual ventilation, natural ventilation + air purifiers, and manual ventilation + air purifiers);	PM_1 - PM_{10}<br>PNC<br>BC	Median concentrations (IQR)<br>Indoor:<br>PNC: < 0.15x10^4 #/cm^3<br>Outdoor:<br>PM_1 - PM_{10}<br>A: 15.8 µg/m^3 (10.9 - 21.0 µg/m^3)	Pacitto et al., 2020

		6 similar air purifiers with 3 specific filters for particles and gaseous pollutants were used;		B: 6.8 μg/m^3 (4.1 - 11.6 μg/m^3)<br>PNC:<br>A: 1.10x10^4 #/cm^3 (0.90 - 1.41x10^4 #/cm^3)<br>B: 1.21x10^4 #/cm^3 (0.93 - 1.51x10^4 #/cm^3)<br>BC<br>A: 2.3 μg/m^3 (1.7 - 3.2 μg/m^3)<br>B: 1.2 μg/m^3 (0.8 - 1.9 μg/m^3)	
2015	Portugal (Oporto)	5 primary schools in Porto;<br>Measurements: October - February 2015, both indoors and outdoors;<br>UFP monitored during the 2nd campaign in 5 school gyms;<br>UFP data resolution of 1 min;	UFP	UFP: 8661 #/cm^3 (5470 - 17330 #/cm^3)	Slezakova et al., 2019 b
2012	Italy (Cassino)	12 elementary school gyms;<br>Each school monitored for 1 school day;<br>Measurements: 5 - 10 min before the beginning of the activities in the gyms until the end of the day (approx. 13:30);	$PM_{2.5}$<br>PM_{10}	$PM_{2.5}$: 17 - 92 μg/m^3<br>PM_{10}: 33 - 204 μg/m^3	Buonanno et al., 2012
2009-2010	Hungary (Debrecen)	3 gymnasiums: 1 preschool, 2 primary schools;<br>1-2 week field campaigns (November 2009 - February 2010);<br>Duration : 8 am to 4-6 pm;	$PM_{2.5}$<br>PM_{10}	$PM_{2.5}$: 12 - 15 μg/m^3<br>PM_{10}: 70 - 180 μg/m^3	Szoboszlai et al., 2011
2005/2006-2008/2009	USA Montana	2 schools gymnasiums (middle and elementary school);<br>Organization: before, during, and after woodstove change;<br>Over 4-year period;<br>24 h samples approximately once per week (during weekday);	$PM_{2.5}$	Winters:<br>$PM_{2.5}$: 21.5 - 30.5 μg/m^3<br>Non-winters:<br>$PM_{2.5}$: 25.6 - 52.7 μg/m^3	Ward et al., 2013
2005-2009	Czech Republic (Prague)	3 elementary school gymnasiums;<br>20 measurement campaigns, each 7 - 11 days long;<br>24 h sampling;	$PM_{2.5-10}$<br>$PM_{1.0-2.5}$	Weekdays:<br>$PM_{2.5-10}$: 13.6 - 24.9 μg/m^3 (1.2 - 9.2 μg/m^3)<br>$PM_{1.0-2.5}$: 3.7 - 7.4 μg/m^3 (0.5 - 17.5 μg/m^3)<br>Weekends:<br>$PM_{2.5-10}$: 1.0 - 1.8 μg/m^3 (0.5 - 4.9 μg/m^3)<br>$PM_{1.0-2.5}$: 0.6 - 1.6 μg/m^3 (0.5 - 5.6 μg/m^3)	Branis and Safranek, 2011
2005-2009	Czech Republic (Prague)	1 elementary school gymnasium;<br>10 campaigns of 7 - 12 days long over 5 years;<br>24 h sampling.	$PM_{2.5}$	Cold seasons:<br>$PM_{2.5}$: 25 ± 12.3 μg/m^3 (6.3 - 62.6 μg/m^3)<br>Warm seasons:<br>$PM_{2.5}$: 17.6 ± 8.6 μg/m^3 (4.5 - 32.1 μg/m^3)	Branis et al., 2009, 2011

OC – Occupied; NOC – Non-Occupied; I – Indoor; O – Outdoor; BC – Black Carbon; UFP – Ultrafine particles; TSP – Total suspended particulate matter; PNC – Particle number concentration; IQR – Interquartile range; n.d. – not detected; n.s. – not specified. Note: Only studies that reported direct values in tables/text were considered.

Table S5 – Summary of protective threshold of analysed pollutants and physical parameters

Pollutants	Units	Protection threshold	Tolerance margin (%)
PM_{10} [a]	[µg/m^3]	50	100
$PM_{2.5}$ [a]	[µg/m^3]	25	100
TVOCs	[µg/m^3]	600	100
CO_2 [a]	[mg/m^3]	2250	30
CO [a]	[mg/m^3]	10	-
T [b]	[º C]	Winter: 16 – 22<br>Summer: 18 – 26	-
HR [b]	[%]	65 ± 10	-

[a]Portaria nº 138-G/2021 de 1 de julho that establishes the requirements for assessing indoor air quality in commercial and service buildings, including protection thresholds, reference conditions and compliance criteria, and the respective methodology for measuring pollutants and monitoring compliance with the approved standards.

[b]Divisão de Infraestruturas Desportivas (DIED), 2013. Ordinance approving the Technical Regulations for Sports Facilities (RTID, in Portuguese).

Table S6 - Spearman correlation coefficients (r_s) for $PM_{2.5}$ and PM_{10} in the different functional spaces of the 8 health clubs (HCs).

Spearman correlation coefficients (r_s)		
	C&B	Studios
HC1	0.879	0.882
HC2	0.911	0.879
HC3	0.830	0.897
HC4	0.957	0.905
HC5	0.949	0.851
HC6	0.892	0.887
HC7	0.961	0.916
HC8	0.951	0.461
Min	0.830	0.461
Max	0.961	0.916
Median	0.909	0.888

Table S7 - Spearman correlation coefficients (r_s) between PNC and other pollutants ($PM_{2.5}$, PM_{10}, TVOCs and O_3) in the different functional spaces of the eight health clubs (HCs).

Spearman correlation coefficients (r_s)									
		C&B				Studios			
		$PM_{2.5}$	PM_{10}	TVOCs	O_3	$PM_{2.5}$	PM_{10}	TVOCs	O_3
HC1	PNC	0.008	-0.031	0.017	-	-0.587	-0.622	-0.128	-
HC2		-0.294	-0.853	0.070	-	0.677	0.665	-0.302	-
HC3		0.393	0.285	0.137	-	0.282	0.218	-0.020	-
HC4		-0.229	-0.153	0.170	-	0.110	0.109	0.252	-
HC5		0.156	0.162	-0.071	-	-0.089	0.026	0.186	-
HC6		-0.267	-0.113	0.215	-	0.229	0.174	-0.613	-
HC7		0.291	0.306	-0.147	-	0.402	0.199	-0.193	-
HC8		0.550	0.502	0.101	-	-0.147	-0.335	0.461	-
Overall		0.264	0.305	0.110	0.077	0.496	0.629	0.269	0.037

Table S8 – Levels of gaseous pollutants (total volatile compounds – TVOCs, ozone – O_3, and carbon dioxide – CO_2) in indoor air of cardio fitness and bodybuilding areas (C&B) and studios (S) of eight health clubs (HC1 – HC8) during occupied periods.

		TVOCs (mg/m^3)		CO_2 (mg/m^3)		O_3 (µg/m^3)	
		Mean (Min - Max)	Median (25 - 75th)	Mean (Min - Max)	Median (25 - 75th)	Mean (Min - Max)	Median (25 - 75th)
HC1	C&B	0.38<br>(0.06 - 2.91)	0.31<br>(0.18 - 0.48)	1453<br>(807 - 2859)	1366<br>(1198 - 1632)	48.71<br>(3.40 - 97.40)	50.31<br>(42.89 - 66.42)
	S	0.34<br>(0.12 - 1.44)	0.27<br>(0.21 - 0.38)	941<br>(122 - 2660)	1043<br>(347 - 1270)	27.2<br>(0.08 - 63.81)	32.34<br>(21.32 - 35.20)
HC2	C&B	0.29<br>(0.01 - 1.39)	0.24<br>(0.19 - 0.35)	1551<br>(795 - 2798)	1471<br>(1279 - 1751)	44.55<br>(0.08 - 72.06)	49.21<br>(34.27 - 50.41)
	S	0.29<br>(0.17 - 0.81)	0.27<br>(0.23 - 0.35)	2589<br>(1092 - 6323)	2036<br>(1680 - 3471)	33.17<br>(0.08 - 57.50)	34.89<br>(21.49 - 41.23)
HC3	C&B	2.38<br>(0.58 - 24.58)	2.41<br>(1.90 - 2.85)	1265<br>(864 - 1839)	1232<br>(1145 - 1363)	54.28<br>(0.08 - 98.17)	60.68<br>(41.78 - 67.22)
	S	7.59<br>(1.07 - 39.67)	5.34<br>(3.60 - 8.19)	1510<br>(977 - 2351)	1505<br>(1285 - 1641)	89.75<br>(63.87 - 119.71)	92.35<br>(82.64 - 93.40)
HC4	C&B	2.12<br>(1.04 - 7.25)	1.83<br>(1.56 - 2.69)	1567<br>(761 - 2924)	1484<br>(1271 - 1743)	34.01<br>(12.33 - 65.06)	38.65<br>(26.35 - 40.71)
	S	4.41<br>(0.98 - 19.05)	3.46<br>(2.88 - 4.80)	2252<br>(1000 - 4031)	2083<br>(1430 - 3000)	36.96<br>(18.18 - 70.71)	32.4<br>(28.06 - 43.42)
HC5	C&B	3.21<br>(0.10 - 10.26)	2.86<br>(2.33 - 4.15)	1587<br>(994 - 3107)	1501<br>(1285 - 1814)	40.85<br>(0.08 - 90.04)	34.33<br>(33.09 - 41.58)
	S	4.01<br>(2.27 - 8.82)	4.02<br>(2.72 - 4.57)	1802<br>(1183 - 2461)	1829<br>(1624 - 2014)	68.87<br>(27.26 - 85.47)	75.65<br>(68.33 - 76.38)
HC6	C&B	1.19<br>(0.10 - 8.98)	0.57<br>(0.25 - 1.74)	981<br>(745 - 6914)	936<br>(878 - 1017)	58.21<br>(0.08 - 116.20)	68.04<br>(43.88 - 69.84)
	S	2.79<br>(0.41 - 12.55)	2.37<br>(0.93 - 3.08)	1249<br>(775 - 1926)	1283<br>(1037 - 1418)	27.38<br>(5.18 - 43.53)	25.49<br>(23.01 - 29.68)
HC7	C&B	2.03<br>(0.14 - 17.08)	1.85<br>(0.77 - 2.25)	2016<br>(1061 - 8296)	1866<br>(1531 - 2214)	47.53<br>(0.24 - 98.38)	51.52<br>(43.73 - 56.56)
	S	3.35<br>(0.96 - 13.81)	2.58<br>(1.59 - 4.56)	2778<br>(1448 - 6780)	2538<br>(2011 - 3255)	28.18<br>(0.08 - 49.06)	31.97<br>(23.03 - 34.13)
HC8	C&B	1.00<br>(0.10 - 4.80)	0.93<br>(0.69 - 1.28)	1306<br>(754 - 2585)	1208<br>(1023 - 1549)	< 51 ppb	< 51 ppb
	S	1.41<br>(0.10 - 7.19)	1.00<br>(0.33 – 2.13)	1422<br>(791 - 3598)	1383<br>(1104 - 1606)	< 51 ppb	< 51 ppb

Table S9 – Levels of gaseous pollutants (total volatile compounds – TVOCs, ozone – O_3, and carbon dioxide – CO_2) in indoor air of cardio fitness and bodybuilding areas (C&B) and studios (S) of eight health clubs (HC1 – HC8) during non-occupied periods.

		TVOCs (mg/m^3)		CO_2 (mg/m^3)		O_3 (µg/m^3)	
		Mean (Min - Max)	Median (25 - 75th)	Mean (Min - Max)	Median (25 - 75th)	Mean (Min - Max)	Median (25 - 75th)
HC1	C&B	0.41 (0.08 - 1.99)	0.42 (0.33 - 0.52)	1109 (799 - 1717)	1060 (1006 - 1183)	52.62 (16.60 - 88.68)	55.87 (48.25 - 58.98)
	S	0.60 (0.08 - 3.16)	0.55 (0.46 - 0.69)	1111 (133 - 2894)	1167 (948 - 1330)	40.13 (0.08 - 69.69)	40.86 (31.85 - 49.14)
HC2	C&B	0.31 (0.01 - 1.61)	0.32 (0.22 - 0.34)	1213 (739 - 3314)	1144 (1084 - 1343)	43.93 (17.67 - 74.99)	45.94 (34.24 - 53.77)
	S	0.28 (0.03 - 1.18)	0.27 (0.23 - 0.30)	1789 (739 - 6740)	1800 (1526 - 2171)	51.46 (0.08 - 85.89)	55.48 (41.31 - 64.43)
HC3	C&B	3.92 (0.58 - 26.54)	3.99 (2.53 - 5.14)	939 (859 - 1285)	942 (914 - 957)	65.88 (13.57 - 103.36)	70.61 (46.29 - 79.84)
	S	5.83 (0.71 - 35.50)	4.09 (3.68 - 5.54)	1166 (851 - 1944)	1169 (1142 - 1174)	94.46 (46.48 - 119.06)	95.10 (88.22 - 95.69)
HC4	C&B	2.48 (1.01 - 33.67)	2.00 (1.90 - 2.68)	979 (765 - 2442)	986 (951 - 1021)	45.36 (5.60 - 68.20)	49.34 (38.55 - 53.79)
	S	4.42 (0.64 - 27.11)	4.36 (3.60 - 5.72)	1501 (760 - 3985)	1510 (1409 - 1567)	44.33 (4.72 - 73.18)	45.82 (33.65 - 57.92)
HC5	C&B	3.71 (0.17 - 31.13)	3.97 (3.02 - 4.42)	1196 (872 - 2291)	1075 (1064 - 1157)	57.88 (0.16 - 75.32)	57.56 (54.40 - 61.80)
	S	2.86 (1.12 - 9.00)	2.93 (2.33 - 3.47)	1475 (1145 - 2570)	1446 (1408 - 1513)	79.51 (0.16 - 97.50)	79.74 (75.52 - 83.73)
HC6	C&B	0.71 (0.10 - 6.42)	0.69 (0.57 - 0.83)	946 (738 - 1204)	972 (924 - 975)	64.87 (21.20 - 88.39)	67.04 (66.56 - 71.89)
	S	3.05 (0.10 - 18.51)	3.55 (2.21 - 4.14)	1022 (774 - 1793)	1015 (952 - 1085)	26.85 (3.80 - 57.37)	25.11 (22.27 - 29.69)
HC7	C&B	1.66 (0.15 - 11.33)	1.49 (0.65 - 2.17)	1697 (1131 - 5005)	1674 (1550 - 1851)	50.28 (13.17 - 80.54)	53.25 (42.14 - 61.72)
	S	5.37 (0.94 - 20.32)	4.37 (3.30 - 5.61)	2122 (1111 - 7272)	2120 (1892 - 2306)	26 (0.08 - 50.26)	30.81 (26.94 - 32.88)
HC8	C&B	0.33 (0.10 - 0.58)	0.34 (0.29 - 0.37)	851 (728 - 1511)	850 (818 - 882)	<51 ppb	<51 ppb
	S	1.7 (0.10 - 12.66)	1.40 (1.10 - 2.51)	1132 (781 - 4268)	1136 (1085 - 1280)	<51 ppb	<51 ppb

Table S10 - Summary of existent studies on gaseous pollutants in sport facilities.

Year of study	Country, city	Study description	Pollutants	Levels	Reference
**Health clubs/ fitness centers**					
2021	Germany (Essen)	Study carried out with the aim of evaluating the feasibility of opening gyms during the COVID-19 blockade period, in order to minimize the spread of the SARS-CoV-2 virus;<br>Study duration: 4 days, May 2021;<br>Users performed 1-hour training sessions, having to leave the premises for 15 minutes, with 19 intervals throughout the day;	CO_2<br>T (ºC)<br>HR (%)	CO_2: 378 ppm (fitness classroom) – 983 ppm (free weight área)<br>T (ºC): 21 - 22.1 ºC<br>HR (%): 48.9 - 53.7 %	Huessler et al., 2022
2020	Netherlands (Eindhoven)	Conducting 2 studies:<br><br>Study 1:<br>Measurements of endogenous (saliva) and exogenous particles (PMtgenerated during physical exercise);<br>STudy carried out in a test room equipped with air conditioning and a ventilator;<br>There was no air supply or exhaust from the room;<br>3 human volunteers who performed 2 sessions of 30 minutes each;<br><br>Study 2:<br>Student Sports Center at Eindhoven University of Technology - Fitness and bodybuilding room;<br>Mechanical mixing ventilation system - fresh air supplied by openings with swirl diffusors in the ceiling;<br>30-minute sessions with 6 different scenarios (ventilation, air cleaning and physical activity);	T (ºC)<br>HR (%)	Study 1:<br>T = 21 ºC<br>HR = 55-65%<br><br>Study 2:<br>T:<br>1: 18.6 – 19.6 ºC<br>2: 19.6 – 19.5 ºC<br>3: 19.6 – 20.7 ºC<br>4: 21 – 20.9 ºC<br>5: 21.3 – 22.2 ºC<br>6: 22.3 – 22.3ºC<br>HR:<br>1: 44.0 – 46.3%<br>2: 46.3 – 44.7%<br>3: 44.8 – 49.5%<br>4: 51.1 – 50.6%<br>5: 44.5 – 49.8%<br>6: 50 – 50%	Blocken et al., 2021
2018-2019	Iran (Tehran)	50 gyms (non-specified) in the city of Tehran;<br>Monitoring carried out between November 2018 and March 2019, between 10 am and 11 pm;<br>Sample collection volume: 10L;	Benzene<br>Toluene<br>Ethylbenzene<br>Xylene<br>T (ºC)<br>HR (%)	Benzene: 75.1 ± 36.2 $\mu g/m^3$<br>Toluene: 34.1 ± 23.8 $\mu g/m^3$<br>Ethylbenzene: 54.8 ± 34.9 $\mu g/m^3$<br>Xylene: 19.5 ± 9.1 $\mu g/m^3$<br>T: 21.71 ºC (18.90 - 25.40 ºC)<br>HR: 73.29% (63 - 81.4%)	Dehghani et al., 2019
2018	Brazil (Santa Catarina)	3 fitness centers (A, B and C);<br>Use of air conditioning;<br>Duration of 3 days a week when with the largest number of users;<br>Data collection day: at 7:30 am at 9:30 pm.	CO_2	CO_2<br>A: 3752.13 ppm (597 - 7533 ppm)<br>Median: 3525.50 ppm<br>B: 1000.44 ppm (461 - 2277 ppm)<br>Median: 914.00 ppm<br>C: 1360.89 ppm (798 - 2286 ppm)	Andrade et al., 2018

				Median: 1364.00 ppm	
2016	Thailand (Nakhon Pathom Province)	3 fitness centers: 2 indoor (municipality and university and complexes) and 1 outdoor open-air facility;<br>Field measurements conducted 3 times (beginning, middle and end of operating hours) over period of 39 days;	T (ºC)<br>RH (%)	T: 24.6 – 34.4 ºC (20.9 - 36.6 ºC)<br>RH: 57.1 – 72.1 % (49.7 - 99.8 %)	Onchang and Panyakapo, 2016
2015	Portugal (Lisbon)	Measurements the concentrations of the pollutants in a monitoring program performed in 63 fitness classes (aerobic (A) and holistic (H));<br>Duration of each class: 45 min;<br>Data logged in 1-minute intervals.	TVOC<br>CH_2O<br>CO_2<br>O_3<br>CO	TVOC<br>A: 0.48 ± 0.42 mg/m^3 (0.01 - 2.5 mg/m^3)<br>H: 0.38 ± 0.23 mg/m^3 (0.16 - 0.85 mg/m^3)<br>CH_2O<br>A: 0.07 ± 0.06 mg/m^3 (0.01 - 0.25 mg/m^3)<br>H: -<br>CO_2<br>A: 1682 ± 599 mg/m^3 (208 - 5964 mg/m^3)<br>H: 1662 ± 376 mg/m^3 (831 - 2406 mg/m^3)<br>O_3<br>A: 0.01 ± 0.054 mg/m^3 (0.005 - 1.9 mg/m^3)<br>H: -<br>CO<br>A: 0.64 ± 0.49 mg/m^3 (0.005 - 2.7 µg/m^3)<br>H: 0.29 ± 0.12 mg/m^3 (0.12 - 0.69 mg/m^3	Ramos et al., 2015
2014	Portugal (Oporto)	4 health clubs<br>Different indoor spaces: cardiofitness/bodybuilding areas + studios group classes;<br>2 clubs with swimming pool;<br>2 clubs with HVAC system ventilation;<br>Continuous measurements (7 days – 24h per day), during 40 days (May - June, 2014).	TVOCs<br>CO_2<br>O_3<br>CO	VOCs<br>HC1: 0.014 - 21.8 mg/m^3<br>(Median: 1.4 mg/m^3)<br>HC2: 0.002 - 20.4 mg/m^3<br>(Median: 1.1 mg/m^3)<br>HC3: 0.073 - 12.4 mg/m^3<br>HC4: 0.003 - 8.0 mg/m^3<br>CO_2: 1558 mg/m^3<br>HC1: 733 - 8122 mg/m^3<br>HC2: 697 - 5299 mg/m^3<br>HC3: 1046 - 7649 mg/m^3<br>HC4: 252 - 49007 mg/m^3<br>O_3: 39.8 - 119 µg/m^3<br>HC1: 20 - 118 µg/m^3<br>HC2: 20 - 1660 µg/m^3<br>HC3: 20 - 1100 µg/m^3<br>HC4: 20 - 2490 µg/m^3<br>CO (median)<br>HC1: 0.181 mg/m^3<br>HC3: 1.26 mg/m^3	Slezakova et al., 2018 b
2014	Portugal (Lisbon)	11 fitness centres with fitness/ bodybuilding area + 2 studios (during classes);	CO<br>CO_2	CO: 0 - 2.6 mg/m^3 (0 - 2.8 mg/m^3)<br>CO_2: 1069 - 4418 mg/m^3 (381 - 5964 mg/m^3)	Ramos et al., 2014

		Short-term (45-60 min) measurements conducted in the most occupied periods (late afternoon/ night);<br>3 fitness centres with continuos measurements (approx. 6 days);<br>9 fitness centres with mechanical ventilations;	VOC<br>O_3<br>T (ºC)<br>RH (%)	VOC: 0 - 3.3 mg/m^3 (0.04 - 3.8 mg/m^3)<br>O_3: 0 - 0.19 mg/m^3 (0 - 0.82 mg/m^3)<br>T: 15 - 25 ºC (14 - 25 ºC)<br>RH: 43 - 86 % (40 - 95 %)	
n.s.	Greece (Athens)	Personal exposure assessment study;<br>Various indoor spaces including 1 gym ;<br>Used TSI Sidepak portable sampling pumps (SP530);<br>Sampler placed at the breathing level of the volunteers (2), for approx. 8h;<br>Duration for 3 consecutive days;<br>Quantification by gravimetric method;	T (ºC)<br>HR (%)	T: 30 ºC<br>HR: 33%	Saraga et al., 2014
**Sport facilities**					
2021	China (Hangzhou)	One badminton hall;<br>To assess characteristics of IAQ in sports spaces for public use, with natural ventilation;<br>Measurements during the wet season;<br>IAQ measurements + surveys;	VOCs<br>CO_2<br>T (ºC)<br>RH (%)	VOCs < 0.1 ppm<br>CO_2: 1000 ppm<br>T (ºC): 25.4 - 27.8°C<br>HR: > 90%	Xie et al., 2021
2019	Japan (Yokohama)	Championship: main stadium and warm-up zone;<br>Monitoring before and during the championship (9-12 May);<br>NO_2, O_3, NO and CO monitored using electrochemical sensors for gas phase pollutants;<br>Temperature (T), relative humidity (RH) monitored; with a laser scattering sensor;<br>Data resolution: 5 min time ;	NO_2<br>O_3<br>CO<br>T (ºC)<br>RH (%)	Median (IQR)<br>Stadium:<br>NO_2: 25.6 - 26.9 µg/m^3 (12.1 - 35.6 µg/m^3)<br>O_3: 84.1 µg/m^3 (57.5 - 109.2 µg/m^3)<br>CO: 424 µg/m^3 (373 - 491 µg/m^3)<br>Warm-up track:<br>NO_2: 26.8 - 31.9 µg/m^3 (10.2 - 44.4 µg/m^3)<br>O_3: 80.5 µg/m^3 (55.9 - 113.2 µg/m^3)	Reche et al., 2020
2017	Poland (Warsaw)	A sports venue;<br>Study carried out inside and outside in a season without heating (May/Jun 2017) and with heating (Oct/Nov 2017);<br>CO_2 levels are related to the type of physical activity and the number of users;<br>The levels of VOCs, NO_2 and SO_2 were influenced by external sources (traffic emissions and fossil fuel combustion);	VOCs<br>CO_2<br>NO_2<br>SO_2	VOCs: 129.4 - 193.3 µg/m^3<br>CO_2<br>I: 761 - 815 ppm<br>O: 521 - 525 ppm<br>NO_2: 20.2 - 21.3 µg/m^3<br>SO_2: 0.9 - 1.4 µg/m^3	Bralewska et al., 2022
2017	Poland (Lublin)	3 fitness facilities;<br>Room types: gym, fitness room for group and individual exercises, women's and men's locker;<br>March, July and September 2017;<br>CO_2 - measurements continuous, 24 hours a day;	CO_2	CO_2: 450–520 ppm<br>CO_2 max: 2864 ppm	Staszowska and Dudzi´nska, 2021

2013	Korea	64 indoor screen golf courses located;<br>Duration: September to November 2013<br>Without the distinction of seasons, at the game rooms (G) and lobbies (L).	CO_2<br>CO<br>NO_2<br>O_3<br>CH_2O<br>TVOCs<br>Rn	CO_2<br>G: 615.9 ppm (320.0 - 934.6 ppm)<br>L: 575.6 ppm (336.3 - 885.3 ppm)<br>CO<br>G: 3.1 ppm (0.6 - 21.7 ppm)<br>L: 2.4 ppm (0.5 - 12.9 ppm)<br>NO_2<br>G: 0.03 ppm (0.03 - 0.06 ppm)<br>L: 0.03 ppm (0.03 - 0.06 ppm)<br>O_3<br>G: 0.005 ppm (0.001 - 0.023 ppm)<br>L: 0.005 ppm (0.001 - 0.025 ppm)<br>HCHO<br>G: 53.8 μg/ m^3 (10.0 - 137.6 μg/m^3)<br>L: 45.5 μg/ m^3 (10.7 - 123.0 μg/m^3)<br>TVOCs<br>G: 186.8 μg/ m^3 (23.4 - 768.0 μg/m^3)<br>L: 179.2 μg/ m^3 (17.6 - 798.9 μg/m^3)<br>Rn<br>G: 17.3 Bq/ m^3 (7.4 - 40.7 Bq/m^3)<br>L: 18.6 Bq/ m^3 (7.4 - 48.1 Bq/m^3)	Goung et al., 2014
2012	Spain<br>(Leon)	University facilities: 1 gymnasium and 1 fronton (court to play paddle ball);<br>1-week consecutive measurements;<br>Weekdays (occupied) vs. background (weekends).	TVOC<br>CO_2<br>CO<br>T (ºC)<br>RH (%)	Gym:<br>TVOC: 82.4 ppb (30 - 2300 ppb)<br>CO_2: 413 ppm (370 - 565 ppm)<br>CO: 0.17 (< DL - 12.6 ppm)<br>T: 21.1 ºC (15.9 - 32.5 ºC)<br>RH: 38.7 % (22 - 49.8 %)<br><br>Fronton:<br>TVOC: 53 ppb (35 - 2318 ppb)<br>CO_2: 468 ppm (397 - 787 ppm)<br>CO: 0.01 (< DL - 2.10 ppm)<br>T: 29 ºC (20.4 - 36.6 ºC)<br>RH: 25.8 % (10.8 - 37.3 %)	Alves et al., 2013
2002	Greece<br>(Athens)	2 athletic halls with different ventilation systems (natural and mechanical);<br>Study period:<br>15/02/2002 – 02/03/2002 (natural ventilation)<br>08/03/2002 – 02/04/2002 (mechanical ventilation);<br>Measurements taken simultaneously at two heights in the halls (at the arena (A) and spectators' seats (S)) and outdoors (O), during event period and no event period;<br>Time data resolution: 15 min - at the arena level;<br>BTX - monitoring (at spectators seating area);	O_3<br>NO<br>NO_2<br>BTX	Naturally ventilation<br><br>Event period<br>O_3<br>A: 8 μg/m^3<br>S: 15 μg/m^3<br>O: 40 μg/m^3<br>NO:<br>A: 23 μg/m^3<br>S: 45 μg/m^3<br>O: 29 μg/m^3	Stathopoulou et al., 2008

NO_2:
A: 43 µg/m^3
S: 61 µg/m^3
O: 44 µg/m^3
Benzene: n.d.
Toluene: 91 µg/m^3
Xylene: 61 µg/m^3
No event period
O_3
A: 14 µg/m^3
S: 17 µg/m^3
O: 55 µg/m^3
NO:
A: 38 µg/m^3
S: 93 µg/m^3
O: 17 µg/m^3
NO_2:
A: 47 µg/m^3
S: 62 µg/m^3
O: 39 µg/m^3
Benzene: n.d.
Toluene: 81 µg/m^3
Xylene: 56 µg/m^3

Mechanical ventilation

Event period
O_3
A: 22 µg/m^3
S: 36 µg/m^3
O: 53 µg/m^3
NO:
A: 16 µg/m^3
S: 39 µg/m^3
O: 14 µg/m^3
NO_2:
A: 41 µg/ m^3
S: 44 µg/m^3
O: 31 µg/m^3
Benzene: 39 µg/m^3
Toluene: 95 µg/m^3
Xylene: 53 µg/m^3

No event period
O_3
A: 24 µg/m^3
S: 37 µg/m^3

				O: 53 µg m^3<br>NO:<br>A: 9 µg/m^3<br>S: 30 µg/m^3<br>O: 10 µg/m^3<br>NO_2:<br>A: 37 µg/m^3<br>S: 39 µg/m^3<br>O: 34 µg/m^3<br>Benzene: 46 µg/m^3<br>Toluene: 79 µg/m^3<br>Xylene: 50 µg/m^3	
n.s.	Malasya<br>(Shah Alam)	1 university gym;<br>Old (22 yrs) airtight sealed building ;<br>Measurements made using a Multiparameter Environmental Monitoring (EVM-7);	CO_2<br>T (ºC)<br>RH (%)	$CO_{2\ Max}$: 510 ppm<br>T: 22.3 - 27.4 (ºC)<br>RH: 55.2 - 68.9 %	Mohd Hashim et al., 2019
**Educational facilities**					
2015	Spain<br>(Barcelona)	2 school gyms (A and B) naturally ventilated;<br>Period: February and April 2015;<br>4 different ventilation strategies:<br>(natural ventilation, manual ventilation, natural ventilation + air purifiers and manual ventilation + air purifiers).	CO_2<br>NO_2	Median concentrations<br>CO_2:<br>A: 400 – 900 ppm<br>B: 420 – 1000 ppm<br>NO_2:<br>A: 10 – 25 ppb<br>B: 20 – 60 ppb	Pacitto et al., 2020

I – Indoor; O – Outdoor; IQR – Interquartile range; DL – detection limit; n.a. – not available. Note: Only studies that reported direct values in tables/text were considered.

Table S11 – Levels of physical parameters (temperature –T, relative humidity – RH) in indoor air of cardiofitness and bodybuilding areas (C&B) and studios (S) of eight health clubs (HC1 – HC8) during occupied periods

		T (ºC)		RH (%)	
		Mean (Min - Max)	Median (25 - 75th)	Mean (Min - Max)	Median (25 - 75th)
HC1	C&B	23.1 (19.9 - 25.7)	23.2 (22.1 - 24.2)	55.8 (42.0 - 73.4)	54.6 (50.2 - 62.8)
	S	21.4 (17.7 - 24.0)	21.8 (20.4 - 22.6)	61.3 (49.7 - 73.2)	60.1 (58.4 - 64.4)
HC2	C&B	21.7 (15.3 - 23.9)	22.1 (21.6 - 22.6)	61.6 (50.3 - 89.1)	58.7 (55.8 - 64.9)
	S	21.2 (17.9 - 23.2)	21.3 (20.2 - 22.5)	68.8 (49.6 - 85.2)	66.6 (65.5 - 75.0)
HC3	C&B	23.6 (21.4 - 25.0)	23.8 (23.0 - 24.2)	50.9 (41.7 - 59.7)	51.2 (46.7 - 55.0)
	S	24.9 (23.6 - 26.1)	24.8 (24.4 - 25.4)	53.8 (48.2 - 61.5)	53.5 (51.4 - 56.0)
HC4	C&B	21.5 (20.0 - 23.9)	21.6 (21.0 - 21.9)	63.3 (52.2 - 70.5)	63.7 (61.2 - 65.9)
	S	22.3 (20.0 - 24.2)	22.5 (21.4 - 23.2)	62.2 (53.9 - 73.5)	61.2 (58.8 - 65.3)
HC5	C&B	21.3 (14.3 - 25.2)	21.9 (21.1 - 22.7)	63.9 (53.4 - 89.7)	61.2 (59.0 - 64.4)
	S	22.3 (17.2 - 24.6)	23.7 (20.5 - 24.2)	62.8 (57.8 - 72.8)	61.5 (59.7 - 66.2)
HC6	C&B	23.5 (18.6 - 28.6)	23.6 (21.7 - 25.0)	52.3 (27.8 - 82.0)	51.4 (45.3 - 56.5)
	S	21.7 (21.0 - 22.3)	21.7 (21.4 - 21.9)	77.8 (63.7 - 87.0)	78.7 (73.9 - 82.6)
HC7	C&B	22.7 (18.1 - 25.9)	22.5 (22.2 - 22.8)	62.4 (48.0 - 77.8)	62.8 (58.5 - 67.1)
	S	21.1 (17.3 - 22.7)	21.3 (20.6 - 21.8)	64.8 (52.8 - 82.3)	62.7 (60.1 - 69.0)
HC8	C&B	17.4 (15.1 - 19.2)	17.4 (16.9 - 18.1)	57.7 (49.1 - 69.8)	57.2 (53.8 - 61.8)
	S	17.9 (14.8 - 21.1)	17.8 (16.7 - 19.3)	56.3 (47.3 - 70.0)	55.1 (51.7 - 60.5)

Table S12 – Levels of physical parameters (temperature –T, relative humidity –RH) in indoor air of cardiofitness and bodybuilding areas (C&B) and studios (S) of eight health clubs (HC1 – HC8) during non-occupied periods

		T (ºC)		RH (%)	
		Mean (Min - Max)	Median (25 - 75th)	Mean (Min - Max)	Median (25 - 75th)
HC1	C&B	22.9 (20.9 - 25.2)	22.8 (22.2 - 23.2)	55.6 (44.1 - 66.1)	56.4 (51.0 - 59.1)
	S	22.5 (18.0 - 24.3)	22.6 (22.2 - 23.0)	59.1 (47.5 - 75.0)	59.2 (58.7 - 59.6)
HC2	C&B	21.0 (15.1 - 23.5)	21.9 (21.6 - 22.6)	62.4 (51.2 - 85.2)	58.4 (57.7 - 63.1)
	S	22.4 (18.0 - 24.6)	22.7 (21.8 - 23.0)	62.7 (46.4 - 87.1)	62.6 (61.0 - 64.0)
HC3	C&B	24.0 (22.3 - 25.4)	24.3 (23.7 - 24.4)	44.8 (41.1 - 58.2)	44.6 (43.5 - 45.9)
	S	24.8 (21.6 - 26.2)	24.9 (24.6 - 24.9)	52.5 (47.5 - 63.2)	52.3 (50.2 - 54.4)
HC4	C&B	22.6 (19.9 - 24.0)	22.7 (22.1 - 23.2)	59.7 (51.8 - 65.8)	60.0 (58.8 - 61.1)
	S	23.1 (19.9 - 24.3)	23.2 (23.1 - 23.3)	59.4 (52.3 - 74.7)	58.2 (57.9 - 60.4)
HC5	C&B	23.7 (15.6 - 24.5)	23.6 (23.4 - 24.2)	59.8 (56.0 - 83.3)	59.0 (58.2 - 61.7)
	S	23.8 (17.0 - 24.8)	24.1 (23.8 - 24.1)	61.7 (56.7 - 73.1)	60.4 (59.4 - 61.3)
HC6	C&B	24.2 (18.9 - 26.0)	24.5 (23.9 - 24.9)	61.9 (38.2 - 78.0)	56.3 (55.0 - 61.8)
	S	21.7 (20.6 - 23.0)	21.7 (21.5 - 21.8)	76.9 (63.5 - 87.0)	78.2 (75.7 - 79.3)
HC7	C&B	22.9 (21.4 - 23.9)	22.9 (22.7 - 23.4)	63.7 (52.0 - 73.3)	63.4 (57.9 - 67.7)
	S	21.3 (16.9 - 22.7)	21.2 (21.1 - 21.3)	62.2 (48.0 - 85.5)	61.9 (60.2 - 63.1)
HC8	C&B	16.3 (15.1 - 18.1)	16.3 (16.2 - 16.4)	65.7 (54.6 - 74.8)	67.3 (64.4 - 68.7)
	S	17.6 (14.8 - 22.0)	17.5 (17.5 - 18.4)	55.2 (46.2 - 71.1)	54.7 (53.5 - 57.8)

Figure S2 - Overall levels of particulate matter at eight health clubs (NOC = non-occupied; OC = occupied period). The horizontal lines represent Portuguese protection threshold (Ordinance nº 138-G/2021) for PM_{10} (50 µg/m^3) and $PM_{2.5}$ (25 µg/m^3): a) PM_{10}; b) $PM_{2.5}$; c) UFP.

a)

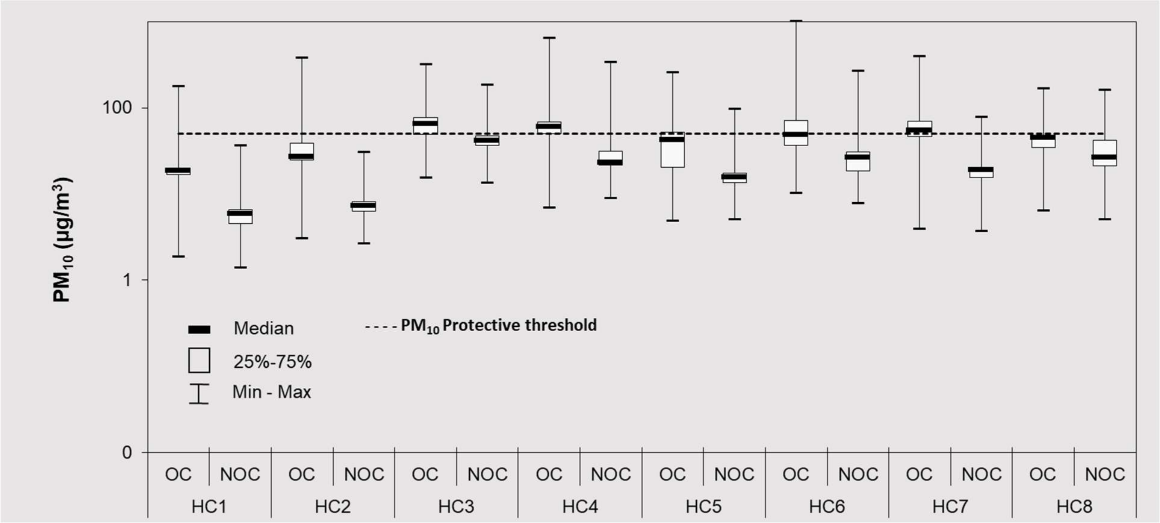

b)

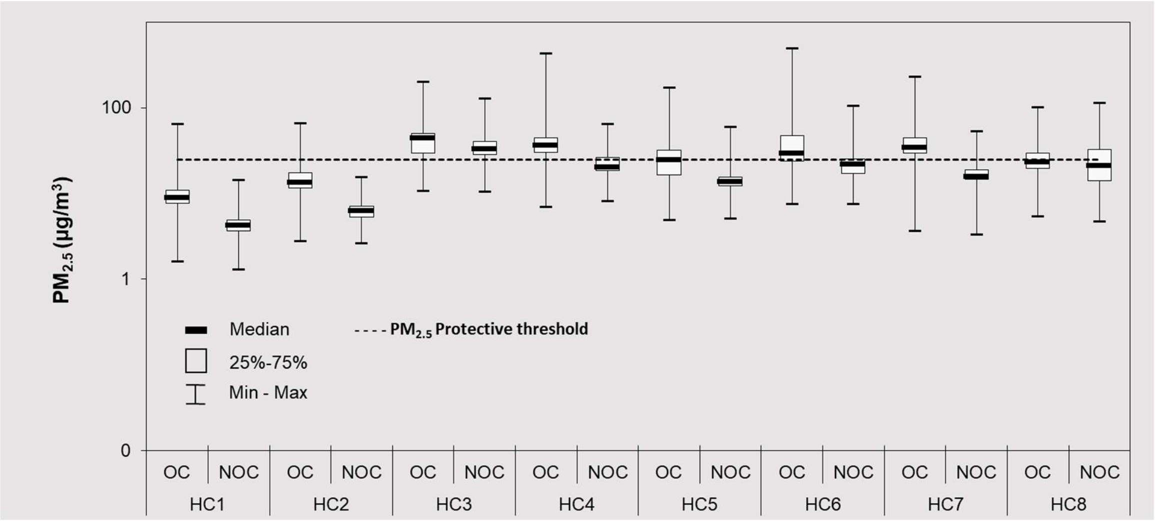

c)

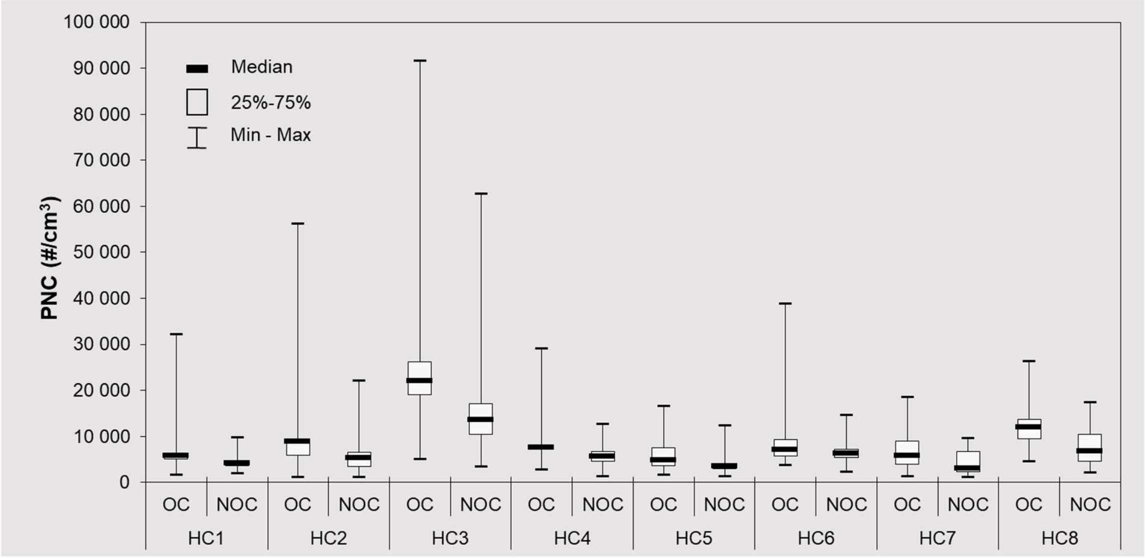

References:

Almeida, S. M., Ramos, C. A., & Almeida-Silva, M. (2015). Exposure and inhaled dose of susceptible population to chemical elements in atmospheric particles. Journal of Radioanalytical and Nuclear Chemistry, 309(1), 309–315. https://doi.org/10.1007/s10967-015-4673-5.

Alves, C.A., Calvo, A.I., Castro, A., Fraile, R., Evtyugina, M., Bate-Epey, E.F., (2013). Indoor air quality in two university sports facilities. Aerosol Air Qual. Res. 13 (6), 1723-1730. https://doi.org/10.4209/aaqr.2013.02.0045.

Alves, C., Calvo, A. I., Marques, L., Castro, A., Nunes, T., Coz, E., & Fraile, R. (2014). Particulate matter in the indoor and outdoor air of a gymnasium and a fronton. Environmental Science and Pollution Research, 21(21), 12390–12402. https://doi.org/10.1007/s11356-014-3168-1.

Amouei Torkmahalleh, M., Kabay, K., Bazhanova, M., Mohiuddin, O., Obaidullah, M., & Gorjinezhad, S. (2018). Investigating the impact of different sport trainings on particulate matter resuspension in a sport center using well-characterized reference instruments and a low-cost monitor. Science of The Total Environment, 612, 957–965. https://doi.org/10.1016/j.scitotenv.2017.08.107.

Andrade, A., Dominski, F.H., Pereira, M.L., Liz, C.M., Buonanno, G. (2018). Fitness centers demonstrate CO2 concentration levels above recommended standards. Acta Scientiarum. Health Sciences, vol. 40. Universidade Estadual de Maringá. ISSN on-line: 1807-8648. https://doi.org/10.4025/actascihealthsci.v40i1.35768.

Blocken, B., van Druenen, T., Ricci, A., Kang, L., van Hooff, T., Qin, P., … Brombacher, A. C. (2021). Ventilation and air cleaning to limit aerosol particle concentrations in a gym during the COVID-19 pandemic. Building and Environment, 193, 107659. https://doi.org/10.1016/j.buildenv.2021.1076599

Bralewska, Rogula-Kozłowska, & Bralewski. (2019). Size-Segregated Particulate Matter in a Selected Sports Facility in Poland. Sustainability, 11(24), 6911. https://doi.org/10.3390/su11246911.

Bralewska, K., & Rogula-Kozłowska, W. (2020). Health exposure of users of indoor sports centers related to the physico-chemical properties of particulate matter. Building and Environment, 106935. https://doi.org/10.1016/j.buildenv.2020.106935.

Bralewska, Rogula-Kozłowska, & Bralewski, (2022). Indoor air quality in sports center: Assessment of gaseous pollutants. Building and Environment, 108589. https://doi.org/10.1016/j.buildenv.2021.108589.

Braniš, M., & Šafránek, J. (2011). Characterization of coarse particulate matter in school gyms. Environmental Research, 111(4), 485–491. https://doi.org/10.1016/j.envres.2011.03.010.

Braniš, M., Šafránek, J., & Hytychová, A. (2009). Exposure of children to airborne particulate matter of different size fractions during indoor physical education at school. Building and Environment, 44(6), 1246–1252. https://doi.org/10.1016/j.buildenv.2008.09.01.

Braniš, M., Šafránek, J., & Hytychová, A. (2011). Indoor and outdoor sources of size-resolved mass concentration of particulate matter in a school gym—implications for exposure of exercising children. Environmental Science and Pollution Research, 18(4), 598–609. https://doi.org/10.1007/s11356-010-0405-0.

Buonanno, G., Fuoco, F.C., Marini, S., Stabile, L., (2012). Particle resuspension in school gyms during physical activities. Aerosol Air Qual. Res. 12 (5), 803-813. https://doi.org/10.4209/aaqr.2011.11.0209.

Castro, A., Calvo, A. I., Alves, C., Alonso-Blanco, E., Coz, E., Marques, L., … Fraile, R. (2015). Indoor aerosol size distributions in a gymnasium. Science of The Total Environment, 524-525, 178–186. https://doi.org/10.1016/j.scitotenv.2015.03.1.

Dehghani, M. H., Norouzian, A., Fazlzadeh, M., & Ghaffari, H. R. (2019). Exposure and risk assessment of BTEX in indoor air of gyms in Tehran, Iran. Microchemical Journal, 150, 104135. https://doi.org/10.1016/j.microc.2019.104135.

Divisão de Infraestruturas Desportivas (DIED), 2013. Portaria que aprova o Regulamento Técnico das Instalações Desportivas (RTID) (in portuguese).

Filipe, T.S., Vasconcelos Pinto, M., Almeida, J., Alcobia Gomes, C., Figueiredo, J.P., Ferreira, A., (2013). Indoor air quality in sports halls. Occupational Safety and Hygiene. In: Proceedings of the International Symposium on Occupational Safety and Hygiene. SHO, pp. 175-179, 2013.

Goung, S. J. N., Yang, J., Kim, Y. S., & Lee, C. M. (2014). A pilot study of indoor air quality in screen golf courses. Environmental Science and Pollution Research, 22(9), 7176–7182. https://doi.org/10.1007/s11356-014-3947-8.

Holmberg, S., and Li, Y. (1998). Modelling of the indoor environment e particle dispersion and deposition. Indoor Air 8, 113–122. https://doi.org/10.1111/j.1600-0668.1998.t01-2-00006.x.

Huessler, E.M., Hüsing, A., Vancraeyenest, M., Jöckel, K.H., Schröder, B. (2022). Air quality in an air ventilated fitness center reopening for pilot study during COVID-19 pandemic lockdown. Building and Environment, 219, 109180. https://doi.org/10.1016/j.buildenv.2022.109180.

Jin, H., He, C., Lu, L., and Fan, J. (2013). Numerical investigation of the wall effect on airborne particle dispersion in a test chamber. Aerosol and Air Quality Research 13, 786–794. https://doi.org/10.4209/aaqr.2012.04.0106.

Kic, P., 2016. Dust pollution in the sport facilities. Agron. Res. 14 (1), 75-81.

Kuskowska, K., Rogula-Kozlowska, W., Rogula-Kopiec, P. (2018). Particulate matter and polycyclic aromatic hydrocarbons in a selected athletic hall: ambient concentrations, origin and effects on human health. E3S Web of Conferences 28, 01020. https://doi.org/10.1051/e3sconf/20182801020.

Mohd Hashim, N.H., Wan Ismail, W.H., Sulaiman, F.R., and Mokhtar, M.A.S. (2019). Determining Indoor Air Quality in Gymnasium for Indoor Recreation Activity at University Teknologi MARA, Shah Alam. IOP Conf. Series: Earth and Environmental Science 385, 012034. https://doi.org/10.1088/17551315/385/1/012034.

Onchang, R., & Panyakapo, M. (2016). The physical environments and microbiological contamination in three different fitness centres and the participants' expectations: Measurement and analysis. Indoor and Built Environment, 25(1), 213–228. https://doi.org/10.1177/1420326x14543209.

Pacitto, A., Amato, F., Moreno, T., Pandolfi, M., Fonseca, A., Mazaheri, M., … Querol, X. (2019). Effect of ventilation strategies and air purifiers on the children's exposure to airborne particles and gaseous pollutants in school gyms. Science of The Total Environment, 135673. https://doi.org/10.1016/j.scitotenv.2019.135673.

Ordinance nº 138-G/2021 de 1 de julho (in portuguese).. Diário da República n.º 126/2021, 2º Suplemento, Série I de 2021-07-01, páginas 2 – 6.

Ramos, C. A., Wolterbeek, H. T., & Almeida, S. M. (2014). Exposure to indoor air pollutants during physical activity in fitness centers. Building and Environment, 82, 349–360. https://doi.org/10.1016/j.buildenv.2014.08.02.

Ramos, C. A., Reis, J. F., Almeida, T., Alves, F., Wolterbeek, H. T., & Almeida, S. M. (2015). Estimating the inhaled dose of pollutants during indoor physical activity. Science of The Total Environment, 527-528, 111–118. https://doi.org/10.1016/j.scitotenv.2015.04.120.

Reche, C., Viana, M., van Drooge, B. L., Fernández, F. J., Escribano, M., Castaño-Vinyals, G., … Bermon, S. (2020). Athletes' exposure to air pollution during World Athletics Relays: A pilot study. Science of The Total Environment, 717, 137161. https://doi.org/10.1016/j.scitotenv.2020.137161.

Rivas, I., Mazaheri, M., Viana, M., Moreno, T., Clifford, S., He, C., Bischof, O.F., Martins, V., Reche, C., Alastuey, A., Alvarez-Pedrerol, M., Sunyer, J., Morawska, L., and Querol, X. (2017). Identification of technical problems affecting performance of DustTrak DRX aerosol monitors. Science of The Total Environment 584-585:849–855. https://doi.org/10.1016/j.scitotenv.2017.01.129.

Saraga, D. E., Volanis, L., Maggos, T., Vasilakos, C., Bairachtari, K., & Helmis, C. G. (2014). Workplace personal exposure to respirable PM fraction: a study in sixteen indoor environments. Atmospheric Pollution Research, 5(3), 431–437. https://doi.org/10.5094/apr.2014.050.

Slezakova, K., Peixoto, C., Oliveira, M., Delerue-Matos, C., Pereira, M. do C., & Morais, S. (2018a). Indoor particulate pollution in fitness centres with emphasis on ultrafine particles. Environmental Pollution, 233, 180-193. https://doi.org/10.1016/j.envpol.2017.10.050.

Slezakova, K., Peixoto, C., Pereira, M. do C., & Morais, S. (2018b). Indoor air quality in health clubs: Impact of occupancy and type of performed activities on exposure levels. Journal of Hazardous Materials, 359, 56–66. https://doi.org/10.1016/j.jhazmat.2018.07.015.

Slezakova, K., Peixoto, C., Carmo Pereira, M. do, & Morais, S. (2019 a). (Ultra) Fine particle concentrations and exposure in different indoor and outdoor microenvironments during physical exercising. Journal of Toxicology and Environmental Health, Part A, 1–12. https://doi.org/10.1080/15287394.2019.1636494.

Slezakova, K., de Oliveira Fernandes, E., & Pereira, M. do C. (2019 b). Assessment of ultrafine particles in primary schools: Emphasis on different indoor microenvironments. Environmental Pollution, 246, 885–895. https://doi.org/10.1016/j.envpol.2018.12.073.

Stathopoulou, O. I., Assimakopoulos, V. D., Flocas, H. A., & Helmis, C. G. (2008). An experimental study of air quality inside large athletic halls. Building and Environment, 43(5), 834–848. https://doi.org/10.1016/j.buildenv.2007.01.026.

Staszowska, A. and Dudzi´nska, M.R. (2021). CHAPTER 18 Indoor air quality in fitness facilities. In book: Advances in Environmental Engineering Research in Poland. Routledge Taylor & Francis Group. https://doi.org/10.1201/9781003171669-18.

Szoboszlai, Z., Furu, E., Angyal, A., Szikszai, Z., & Kertész, Z. (2011). Investigation of indoor aerosols collected at various educational institutions in Debrecen, Hungary. X-Ray Spectrometry, 40(3), 176–180. https://doi.org/10.1002/xrs.1323.

Ward, T. J., Palmer, C. P., Hooper, K., Bergauff, M., & Noonan, C. W. (2013). The impact of a community–wide woodstove changeout intervention on air quality within two schools. Atmospheric Pollution Research, 4(2), 238–244. https://doi.org/10.5094/apr.2013.025.

Weinbruch, S., Dirsch, T., Kandler, K., Ebert, M., Heimburger, G., & Hohenwarter, F. (2012). Reducing dust exposure in indoor climbing gyms. Journal of Environmental Monitoring, 14(8), 2114. https://doi.org/10.1039/c2em30289f.

Xie, R., Xu, Y., Yang, J., & Zhang, S. (2021). Indoor air quality investigation of a badminton hall in humid season through objective and subjective approaches. Science of The Total Environment, 771, 145390. https://doi.org/10.1016/j.scitotenv.2021.145390.

Žitnik, M., Bučar, K., Hiti, B., Barba, Ž., Rupnik, Z., Založnik, A., … Žibert, J. (2015). Exercise-induced effects on a gym atmosphere. Indoor Air, 26(3), 468–477. https://doi.org/10.1111/ina.12226.
